# Improving Machine Learning Classification Predictions through SHAP and Features Analysis Interpretation

**DOI:** 10.1021/acs.jcim.5c02015

**Published:** 2025-10-20

**Authors:** Leonardo Bernal, Giulio Rastelli, Luca Pinzi

**Affiliations:** † Department of Life Sciences, 9306 University of Modena and Reggio Emilia, Via Giuseppe Campi 103, 41125 Modena, Italy; ‡ Clinical and Experimental Medicine PhD Program, University of Modena and Reggio Emilia, Modena 41125, Italy

## Abstract

Tree-based machine learning (ML) algorithms, such as Extra Trees (ET), Random Forest (RF), Gradient Boosting Machine (GBM), and XGBoost (XGB) are among the most widely used in early drug discovery, given their versatility and performance. However, models based on these algorithms often suffer from misclassification and reduced interpretability issues, which limit their applicability in practice. To address these challenges, several approaches have been proposed, including the use of SHapley Additive Explanations (SHAP). While SHAP values are commonly used to elucidate the importance of features driving models’ predictions, they can also be employed in strategies to improve their prediction performance. Building on these premises, we propose a novel approach that integrates SHAP and features value analyses to reduce misclassification in model predictions. Specifically, we benchmarked classifiers based on ET, RF, GBM, and XGB algorithms using data sets of compounds with known antiproliferative activity against three prostate cancer (PC) cell lines (*i.e.*, PC3, LNCaP, and DU-145). The best-performing models, based on RDKit and ECFP4 descriptors with GBM and XGB algorithms, achieved MCC values above 0.58 and F1-score above 0.8 across all data sets, demonstrating satisfactory accuracy and precision. Analyses of SHAP values revealed that many misclassified compounds possess feature values that fall within the range typically associated with the opposite class. Based on these findings, we developed a misclassification-detection framework using four filtering rules, which we termed “RAW”, SHAP, “RAW OR SHAP”, and “RAW AND SHAP”. These filtering rules successfully identified several potentially misclassified predictions, with the “RAW OR SHAP” rule retrieving up to 21%, 23%, and 63% of misclassified compounds in the PC3, DU-145, and LNCaP test sets, respectively. The developed flagging rules enable the systematic exclusion of likely misclassified compounds, even across progressively higher prediction confidence levels, thus providing a valuable approach to improve classifier performance in virtual screening applications.

## Graphical abstract

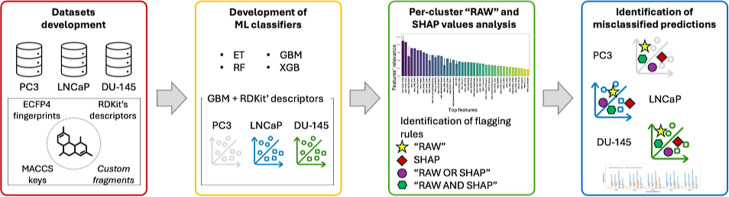

## Introduction

In recent years, artificial intelligence (AI) and in particular machine learning (ML) have gained significant attention in drug discovery.^1,2^ These approaches have enabled the exploration of extensive chemical spaces and accelerated the development of new therapeutic candidates, from initial compound screenings^3–5^ and bioactivity prediction^6^ to the optimization and prioritization of therapeutic leads.^7–9^ Fueled by the increasing availability of large data sets, ML approaches have also contributed to significantly reduce experimental costs and to accelerate timelines traditionally associated with discovery and development,^10^ thereby demonstrating their growing importance in both pharmaceutical industry and academic research.^11^

Despite their utility, several challenges often hamper the application of ML models in drug discovery,^12^ especially during the early stages of virtual screening campaigns. Among these, model interpretability remains a key challenge,^12^ especially for complex architectures based on deep neural networks, which often provide outputs with limited interpretability and understanding of their inner functioning.^13–15^ Such a limitation complicates the translation of model predictions into actionable chemical insights, hampering rational chemical optimization of candidate molecules. To address these limitations, novel deep learning models have been introduced. However, the lack of large, high-quality training data sets containing bioactivity annotations for molecules, biological targets, or cell lines often results in overfitting, generalization issues, and reduced chemical insights.^16^ These issues limit their application in early virtual screening campaigns and *in silico* phenotypic screenings. Conversely, simpler tree-based ML algorithms remain widely used due to their lower implementation complexity and inherent prediction interpretability,^17,18^ which facilitate the understanding of the models’ predictions and provide clearer insights from their output.

Despite these advantages, model prediction outputs often contain false positives and false negatives, which can undermine the efficiency of the experimental validation. To mitigate this issue, many studies rely on probability-based confidence thresholds (*e.g.*, the *predict_proba* function available in SciKit-Learn) to filter uncertain predictions. While effective in reducing globally uncertain classifications, such strategies inherently trade off predictive coverage, often discarding a substantial fraction of compounds without resolving the problem of structurally inconsistent or locally misclassified predictions. This limitation highlights that reliance on global probability thresholds alone is insufficient to detect locally misclassified compounds, particularly when the classifier confidence is artificially high. Besides, explainable artificial intelligence (xAI), particularly SHapley Additive exPlanations (SHAP),^19^ has gained significant attention as a powerful tool for addressing model interpretability challenges. SHAP algorithms employ cooperative game theory principles to quantify the contribution of each feature to the predictions made by ML models, irrespective of model complexity.^19^ In particular, SHAP values provide an objective quantification of a model’s prediction based on its input features, thereby offering clear and interpretable insights into the rationale behind model outputs. In drug discovery contexts,^20–22^ SHAP has been successfully employed to rationalize activity prediction models, prioritize molecular features, and derive mechanistic insights that support rational chemical and biological interpretation.^21,23–28^ However, these applications have primarily focused on retrospective interpretability rather than the prospective reduction of prediction errors. Beyond drug discovery, SHAP has also been explored in other domains for feature selection in both classification and regression tasks,^29,30^ and more recently, in combination with complementary techniques for model development.^31,32^ Despite its potential, the integration of SHAP as a practical tool for actively identifying and filtering out unreliable predictions in virtual screening workflows has not yet been systematically explored. Given that SHAP values quantify feature-level contributions for individual predictions, their analyses can help to identify unexpected or misspecified signals potentially driving misclassifications.

Building on these premises, the present study aims at bridging these gaps by developing a novel framework that integrates raw feature value analysis with SHAP-derived feature contributions to detect and flag potentially misclassified compounds. By combining adaptive, cluster-specific thresholds with both “RAW” and SHAP-informed rules, this approach advances beyond traditional confidence-based filtering methods, offering a computationally efficient, interpretable, and deployable strategy to enhance the robustness of ML classifiers in virtual screening applications.

To this aim, we first developed and systematically compared machine learning classifiers based on routinely employed tree-based algorithms, namely, Extra Trees (ET),^33^ Random Forest (RF), Gradient Boosting Machines (GBM),^34–36^ and XGBoost (XGB).^37^ Models were trained on curated data sets of ChEMBL compounds with experimentally validated antiproliferative activity against LNCaP, DU-145, and PC3 prostate cancer (PC) cell lines. The ML pipeline incorporated rigorous data preprocessing steps, including data curation, stratified train/test splitting, and recursive feature elimination (RFE), to retain only the most informative descriptors and ensure robust and generalizable predictive performance. Different types of features were used to encode the properties and structural details of ChEMBL compounds. These include: (i) RDKit’s molecular descriptors,^38^ offering a broad range of physicochemical and topological properties across chemical space; (ii) MACCS Keys,^39^ which encode for the presence of predefined dictionary of substructures; (iii) ECFP4 fingerprints,^40^ providing detailed atom-centered topological information through circular substructures, and; (iv) custom fragment-based molecular representations, designed to encode larger chemical substructures relevant to biological activity specific for LNCaP, DU-145, and PC3 data sets. Analyses of the SHAP values, optimized for tree-based algorithms, were then performed to systematically assess features' importance within the developed ML models. Notably, several features from models trained using ECFP4 fingerprints and custom fragments were found to contribute negligibly to models’ predictions. Based on these findings, we devised a cluster-based misclassification-detection framework applied to the best-performing classifiers. This approach successfully identified several misclassified compounds whose SHAP values and raw molecular properties (hereafter referred to as “RAW”) fell within the distribution ranges observed for the opposite class. Statistical analyses conducted on correctly classified compounds enabled the definition of *ad hoc* thresholds for detecting potentially incorrect predictions. Given that false positive and false-negative predictions often populate results of early virtual screening and phenotypic screening campaigns performed with ML models, our framework offers a practical strategy for flagging and eliminating such instances.

The results presented in this study demonstrate the utility of integrating model evaluation, explainability, and decision support to improve the reliability of ML-driven predictions. Importantly, the application of explainable AI not only improved transparency in models’ prediction but also offered insights for model refinement, thereby reducing the trial-and-error process typically associated with algorithm and parameters optimization. Overall, the proposed filtering strategy represents a valuable complement to standard classification workflows and holds promise for broad application in virtual screening contexts.

## Results and Discussion

### Generation of the Data Sets of Features for the Development of Machine Learning Classifiers

In this study, four tree-based ML models based on ET, RF, GBM, and XGB algorithms were developed on the PC3, DU-145, and LNCaP data sets (see Supporting File 1), by using four sets of molecular features. In particular, the analyses focused on RDKit’s molecular descriptors, MACCS keys, and ECFP4 fingerprints, which are among the most commonly employed types of features in cheminformatics-based ML workflows.

In addition, novel data set-specific fingerprints named “*custom-fragments*”, *i.e.*, molecular fragments generated through the application of established fragmentation approaches followed by statistical analysis and filtering (see Experimental Methods below), were also devised and applied. Collectively, these types of features enabled a comprehensive representation of the compounds across multiple levels of chemical abstraction, thereby facilitating robust and comparative models’ development for activity prediction. RDKit’s molecular descriptors were selected as they provide chemically meaningful representations of a wide array of physicochemical, structural, and topological molecular attributes. To facilitate the interpretation of their impacts on predictions, investigated molecular descriptors were carefully categorized based on their chemical significance (Table S1). Besides, MACCS keys were also included in the analyses given their simplicity and interpretability; they are one of the most popular chemical representations, being extensively applied in diverse chemoinformatics and ML contexts.^39^ This type of molecular fingerprints is composed of 166 predefined binary descriptors encoding the presence or absence of specific chemical substructures.^39^ After removal of patterns reported in Table S2 (see Experimental Methods), 154 MACCS keys remained, all of which were consistently identified across the PC3, DU-145, or LNCaP data sets (*i.e.*, Table S3). This observation suggests that MACCS keys represent frequently occurring molecular patterns among compounds tested for antiproliferative effects on these prostate cancer cell lines.

Extended-Connectivity Fingerprints (ECFP4) were also used to encode features present in molecules of the PC3, DU-145, and LNCaP data sets, considering their widespread adoption in ML applications due to their effective balance between chemical specificity and computational efficiency.^40^ ECFP4 fingerprints allow encoding atom-centered circular environments as binary fingerprints, at different bit length (*i.e.*, precision) and radius considered (*i.e.*, number of atoms encoded by a single bit).^40^ A number of studies employing ECFP4 fingerprints in ML studies have been reported so far.^21,41,42^ However, most of them has focused on data sets of molecules with data on biological targets and/or closely related chemical classes. In contrast, few investigations have employed ECFP4 fingerprints in phenotypic screenings from large, heterogeneous antiproliferative activity data sets. Recent studies have highlighted intrinsic limitations of ECFP4 fingerprints in ML modeling; these include, for example, their low sensitivity to tautomeric variations,^43^ bit collisions, and sparse vector representations.^44^ Bit collisions, in particular, pose a challenge when the fingerprint’s size (*i.e.*, the number of bits in the fingerprints) is lower with respect to that of the number of substructures resulting by the data set’s compounds. Under such conditions, structurally distinct fragments may be mapped to the same fingerprints’ bit, thereby introducing ambiguity that can compromise both model interpretability and predictive accuracy. While the bit collisions issue is significantly mitigated when performing analyses in reduced chemical spaces,^45,46^ it cannot be neglected when performing in silico phenotypic screenings, which are generally performed from data sets with much broader chemical diversity, as in the cases of the PC3, DU-145, and LNCaP set of compounds.^45,46^ To assess the potential impact of bit collisions in our study, extensive sampling was performed across the PC3, DU-145, and LNCaP data sets and analyzed as described in the Experimental Methods section. In particular, we identified the unique ECFP4-derived features encoded within each sampled subset and calculated the average number of bit collisions per data set. A pronounced increase in bit collisions was observed with increasing data set’s size across all three cell lines (Table 1).

**Table 1:** ECFP4 Bit Collisions Identified in Each Randomly Sampled Fraction of the Data Sets

Data set	Fraction of randomly sampled data set	N° of compounds in the sampled fraction	Averaged bit collision in the sampled fractions^a^
PC3	0.2	2101	20.08 ± 6.44
	0.4	4202	27.79 ± 8.03
	0.6	6303	33.91 ± 9.24
	0.8	8404	38.33 ± 10.2
	1.0	10505	42.17 ± 10.81
DU-145	0.2	1800	16.51 ± 6.96
	0.4	3601	22.58 ± 7.94
	0.6	5401	27.32 ± 8.63
	0.8	7202	30.93 ± 9.23
	1.0	9004	33.82 ± 3.51
LNCaP	0.2	600	7.87 ± 3.51
	0.4	1200	10.98 ± 4.47
	0.6	1800	13.10 ± 5.02
	0.8	2400	14.66 ± 5.46
	1.0	3002	16.19 ± 5.81

a standard deviation is also reported.

This was particularly evident in the PC3 and DU-145 data sets (Figure 1), which present a higher variability in their chemical spaces (Figure S1).

**Figure 1:**
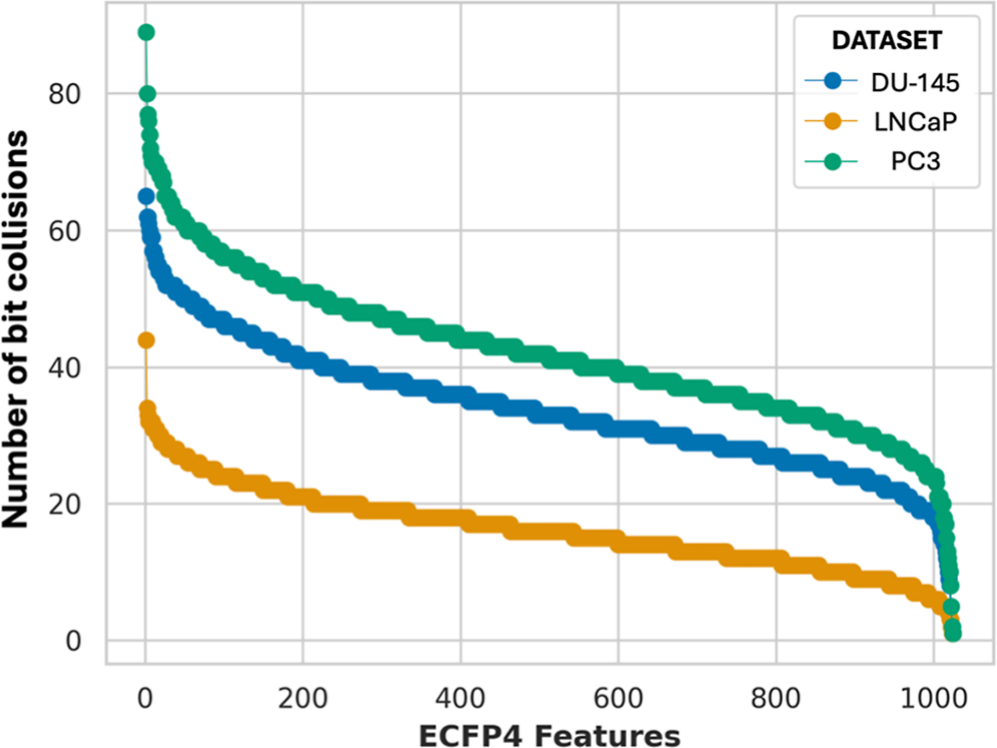
Number of bit collisions in the PC3, DU-145, and LNCaP data sets. Bit collisions are ordered according to their descending frequency.

To quantify this relationship, both Pearson’s and Spearman’s correlation analyses were performed, confirming the robustness and statistical significance of the observed trend. The resulting correlation coefficients were 0.97 (*p* = 1.49 × 10^–9^) and 0.94 (*p* = 1.05 × 10^–^7), respectively, indicating a strong and consistent positive correlation between data set size and fingerprint collision frequency. In addition to MACCS keys and ECFP4 fingerprints, we implemented a tailored fragmentation-based molecular featurization strategy (hereafter referred to as “*custom-fragments*”), derived from multiple scaffold decomposition methodologies, including Bemis-Murcko, RECAP, BRICS, and a ring-specific detachment method.^47–49^ Most studies developing machine learning models based on molecular fragments uses only a single fragmentation method to generate molecular features. To some extent, such an approach might not comprehensively describe molecular features of the compounds in the data set. In this study, we implemented an approach that combines multiple fragmentation strategies to account for such a potential issue. In particular, we included Bemis-Murcko decomposition as it is based on the isolation of the core scaffold from a molecule, removing distal molecular decorations; RECAP and BRICS decomposition methods systematically fragment molecules based on specific chemical rules and bond patterns. Moreover, an additional fragmentation method that isolates ring structures from the remaining scaffold was also implemented. To perform the molecular decompositions and subsequent encoding, custom Python scripts implemented with the RDKit library were developed. Fragment substructures were encoded as SMARTS patterns, ensuring the preservation of aromaticity, stereochemistry, and protonation states. Initial fragmentation yielded a large number of substructures (Table S4). To mitigate potential issues deriving from the use of sparse fingerprints in molecular featurization, only fragments occurring in more than 20 molecules per data set were used to generate molecular fingerprints for ML training. Such a filtering approach allowed us to obtain 1105, 1423, and 1043 unique fragments from the analyses on the PC3, DU-145, and LNCaP data sets, respectively (Table S4). Remarkably, only 104 fragments (around 10%) were found to be in common across all three cell lines, including low decorated structural elements and common functional groups (Table S5). These include, for example, chlorine, hydroxyl, phenyl groups, and commonly employed linkers used in medicinal chemistry (Table S5). Conversely, the low degree of fragment overlaps among PC3, DU-145, or LNCaP data sets suggests the existence of cell line-specific structural patterns associated with antiproliferative activity, whose analysis goes beyond the aim of this study.

### Models Training Performances and Statistical Analysis

The training approach employed in this study was carefully designed to ensure consistency and robustness across all machine learning models that were developed. In particular, each data set was first subjected to a custom clustering-based splitting procedure (see Experimental Methods section). This clustering strategy allowed the preservation of both the chemical diversity and the distribution of activity classes across the training and test sets, thereby enabling more reliable comparisons across the developed models, irrespective of the molecular featurization method or algorithm applied. Afterward, a standardized pipeline was designed and applied for training ML classifiers as follows. Initial hyperparameters' tuning was performed *via* *GridSearch* with 10-fold stratified cross-validation, as described in the Experimental Methods section. This was followed by Recursive Feature Elimination with 10-fold stratified cross-validation (RFEcv) to remove redundant or non-informative features. Subsequently, a second round of hyperparameters' optimization was performed to tune and improve models’ performance on features resulting from the RFEcv. Four different types of tree-based algorithms, namely, ET, RF, GBM, and XGB, were employed for the development of classifiers. Performance metrics at the cross-validation fold level were systematically recorded during training to enable robust statistical comparison. To facilitate models’ interpretability, SHapley Additive exPlanations were computed on the test set with the *Tree.Explainer* module of the SHAP Python library (version 0.48.0).^50^ Mean absolute SHAP values were calculated to assess the relative importance of features and to identify consistent trends across models and cell lines, where applicable. The final hyperparameters' configuration and the number of features retained after RFEcv are summarized in Table S6.

Analyses of the molecular features retained on the developed machine learning models revealed a consistently low number of MACCS key features after RFEcv (*i.e.*, less than 50 features for 10 out of 12; Table S6). This finding suggests that only a limited subset of MACCS features provides relevant information across all the employed data sets and tree-based algorithms employed. This is consistent with the inherently constrained chemical diversity encoded by the MACCS keys. In contrast, models based on *custom-fragment* and ECFP4 fingerprints retained a substantially higher number of features (*i.e.*, more than 500 features for 10 out of 12 ECFP4 fingerprints models, and all developed *custom-fragment* classifiers; Table S6), reflecting the superior capacity of these types of molecular representations to capture the structural complexity and diversity present in the data sets. Despite that ECFP4 fingerprints can provide insights into the chemical patterns guiding compounds’ class assignment in ML models, they very often present explainability issues, hampering their use in medicinal chemistry. Conversely, RDKit’s descriptors exhibited intermediate and more variable numbers of retained features across data sets (*i.e.*, more than 80 features for 11 out of 12; Table S6), in line with their broader range of chemical representation and interpretability (Table S7). Of note, the majority of RDKit’s descriptors commonly retained by ML models trained on LNCaP, PC3, and DU-145 data sets belong to the “Surface Area & VSA (MOE-type)” (*e.g.*, “SlogP_VSA”, “PEOE_VSA”, and “SMR_VSA”) and “Topological Descriptors” (*e.g.*, “Avg_Ipc”, “BalabanJ”, “BertzCT”, “Chi0n”, “Chi4n”, “Chi4v”, and “HallKierAlpha”) classes (as defined in Table S1), suggesting that surface partitioning and branching/topological motifs are particularly informative (Table S7). Besides these descriptors, in LNCaP models, RDKit’s “3D Geometric/Shape Descriptors” (*e.g.*, “3DPMI1” and “3d_SpherocityIndex”) and “Constitutional Descriptors” (*e.g.*, “fr_allylic_oxid”, “fr_ketone”, and “fr_NH0”), which encode for molecular sphericity and the presence of specific substructures, respectively, resulted to be particularly important for models’ development. In contrast, “Constitutional Descriptors”, such as “fr_pyridine”, “NumAliphaticHeterocycles”, and “NumHAcceptors”, resulted to be mostly important for PC3 and DU-145 models (Table S7). Indeed, a significantly higher number of “Constitutional Descriptors” were retained in DU-145 classifiers (Table S7), suggesting that compositional/size features have more importance in the predictive performance for DU-145 than for the other two cell lines. All developed classifiers did not retain “BCUT2D” features, suggesting a limited informative content of these descriptors in the context of these PC cell lines studied. It is noteworthy that key hyperparameters, such as the number of estimators, tree depth, and minimum samples for splitting and leaf formation, remained largely consistent across all algorithms (Table S6). To some extent, this suggests that these values may serve as reasonable starting points for future optimization procedures. Nonetheless, final hyperparameter settings should still be tailored to the specific characteristics of each data set through targeted tuning. Subsequently, performance metrics obtained from the test sets of all trained models were compared to assess and benchmark their prediction performances (Table 2).

**Table 2:** Performance Metrics Obtained on Their Respective Test Sets for the Developed ML Models^a^

Data set	Type of features	Algorithm	Accuracy	MCC	Precision	Recall	ROC-AUC	F1-score
DU-145	ECFP4-based fragments	ET	0.74	0.48	0.80	0.71	0.82	0.75
	*custom-fragments*		0.66	0.33	0.74	0.60	0.74	0.66
	MACCS-based fragments		0.73	0.43	0.73	0.79	0.79	0.76
	RDKit’s descriptors		0.80	0.60	0.80	0.86	0.88	0.83
	ECFP4-based fragments	GBM	0.79	0.58	0.80	0.84	0.87	0.82
	*custom-fragments*		0.69	0.38	0.68	0.86	0.77	0.76
	MACCS-based fragments		0.74	0.47	0.77	0.77	0.81	0.77
	**RDKit’s descriptors**		**0.80**	**0.60**	**0.81**	**0.85**	**0.88**	**0.83**
	ECFP4-based fragments	RF	0.80	0.59	0.83	0.81	0.87	0.82
	*custom-fragments*		0.67	0.34	0.73	0.64	0.76	0.68
	MACCS-based fragments		0.73	0.46	0.78	0.73	0.81	0.75
	RDKit’s descriptors		0.80	0.59	0.83	0.81	0.87	0.82
	ECFP4-based fragments	XGB	0.81	0.61	0.82	0.85	0.88	0.83
	*custom-fragments*		0.73	0.44	0.74	0.79	0.81	0.76
	MACCS-based fragments		0.71	0.41	0.73	0.76	0.77	0.75
	RDKit’s descriptors		0.78	0.55	0.78	0.84	0.86	0.81
LNCaP	ECFP4-based fragments	ET	0.77	0.54	0.89	0.75	0.87	0.81
	*custom-fragments*		0.68	0.44	0.90	0.58	0.81	0.71
	MACCS-based fragments		0.78	0.55	0.90	0.75	0.87	0.82
	RDKit’s descriptors		0.81	0.59	0.88	0.81	0.90	0.85
	ECFP4-based fragments	GBM	0.81	0.58	0.84	0.88	0.89	0.86
	*custom-fragments*		0.76	0.46	0.79	0.86	0.83	0.82
	MACCS-based fragments		0.79	0.53	0.83	0.86	0.87	0.85
	**RDKit’s descriptors**		**0.84**	**0.62**	**0.84**	**0.92**	**0.91**	**0.88**
	ECFP4-based fragments	RF	0.71	0.44	0.86	0.67	0.79	0.76
	*custom-fragments*		0.69	0.44	0.89	0.60	0.80	0.72
	MACCS-based fragments		0.72	0.46	0.87	0.69	0.83	0.77
	RDKit’s descriptors		0.81	0.59	0.88	0.84	0.90	0.86
	ECFP4-based fragments	XGB	0.83	0.60	0.85	0.89	0.90	0.87
	*custom-fragments*		0.77	0.48	0.80	0.88	0.84	0.84
	MACCS-based fragments		0.73	0.38	0.78	0.82	0.77	0.80
	RDKit’s descriptors		0.83	0.62	0.85	0.91	0.91	0.88
PC3	ECFP4-based fragments	ET	0.79	0.58	0.86	0.76	0.88	0.81
	*custom-fragments*		0.64	0.34	0.81	0.50	0.74	0.62
	MACCS-based fragments		0.78	0.56	0.84	0.76	0.86	0.80
	RDKit’s descriptors		0.79	0.58	0.86	0.76	0.88	0.81
	ECFP4-based fragments	GBM	0.80	0.59	0.81	0.86	0.88	0.84
	*custom-fragments*		0.67	0.31	0.68	0.85	0.75	0.75
	MACCS-based fragments		0.82	0.62	0.82	0.88	0.89	0.85
	**RDKit’s descriptors**		**0.80**	**0.59**	**0.81**	**0.87**	**0.88**	**0.84**
	ECFP4-based fragments	RF	0.76	0.55	0.89	0.68	0.87	0.77
	*custom-fragments*		0.65	0.36	0.81	0.52	0.76	0.63
	MACCS-based fragments		0.78	0.55	0.83	0.77	0.86	0.80
	RDKit’s descriptors		0.80	0.59	0.85	0.80	0.88	0.82
	ECFP4-based fragments	XGB	0.83	0.64	0.83	0.89	0.91	0.86
	*custom-fragments*		0.73	0.43	0.73	0.84	0.81	0.78
	MACCS-based fragments		0.79	0.56	0.8	0.86	0.86	0.83
	RDKit’s descriptors		0.78	0.54	0.78	0.86	0.86	0.82

a Best-performing models selected for further analyses are highlighted in bold.

Clear trends emerged upon analysis of the predictive metrics across the developed models. Models built using *custom-fragments* features, despite providing high chemical specificity and interpretability, exhibited comparatively lower predictive performance, with Matthews Correlation Coefficient (MCC) and F1-score values consistently below 0.45 and 0.8, respectively (Table 2). Models developed on MACCS keys demonstrated moderate performance, with MCC values ranging from 0.46 to 0.53 across the PC3, DU-145 or LNCaP data sets (Table 2), reflecting the generic yet less chemically detailed nature of this type of representation. In contrast, ML models based on ECFP4 fingerprints provided overall among the most favorable prediction performances, with MCC and F1-score values ranging from 0.58 to 0.64 and 0.76 to 0.86, respectively (Table 2). However, these models are known to suffer from limited interpretability, particularly due to the presence of bit collisions, which are consistently amplified in larger data sets (*vide supra*) and the high structural diversity of the chemical space represented (Figure S1). Moreover, features mapped by ECFP4 fingerprints are less easy-to-understand compared with those from *custom-fragments* and MACCS keys. Models based on RDKit’s descriptors consistently achieved high prediction performances, with MCC, and F1-score values ranging from 0.59 to 0.62 and 0.82 to 0.88, respectively (Table 2). Nevertheless, the abstract nature of some RDKit’s features may limit their direct mapping to interpretable structure–activity relationships. Interestingly, these models offer a reasonable trade-off between Accuracy and Precision (both around 0.8; Table 2), with predictions being slightly more accurate in detecting compounds that belong to the “active” class (Recall ranged up to 0.92; Table 2). While these findings might derive from the presence of a higher number of “active” compounds in the curated data sets (Figure S2), they also suggest that integrating models with approaches able to reduce false-positive and false-negative predictions might provide significant advantages in virtual screenings. The best-performing models according to MCC and F1-score metrics predominantly derived from ECFP4 fingerprints and RDKit’s descriptors (Table 2). Within the DU-145 data set, the best results were obtained using ET-RDKit and GBM-RDKit combinations, both achieving a MCC of 0.60 and a F1-score of 0.83 (Table 2). For the PC3 data set, the XGB-ECFP4 model achieved the highest MCC (0.64) and a F1-score of 0.86, followed by GBM-MACCS (MCC = 0.62; F1-score = 0.85) and GBM-RDKit (MCC = 0.59; F1-score = 0.84). In the LNCaP data set, RDKit-based models provided the best prediction performances, with both GBM-RDKit and XGB-RDKit achieving an MCC of 0.62 and F1-score of 0.88 (Table 2).

Cross-validation metrics from the training folds were also evaluated to assess the robustness, reliability, and potential overfitting of the developed models. This comparative analysis (Figure S3) confirmed the overall lower performances of models trained on *custom-fragments* across PC3, LNCaP, and DU-145 data sets, likely reflecting their dependency on fragments coverage, which is constrained by the chemical diversity of each data set. However, these models exhibited reduced susceptibility to overfitting compared to those built using RDKit’s molecular descriptors (Figure S3). XGB-based models generally displayed more robust predictions across all types of features, compared with those based on ET and RF. Indeed, the prediction performance of these latter types of algorithms resulted in being more dependent on the employed types of molecular features (Figure S3). Performance variability observed among different types of features and algorithms highlights the importance of context-specific model selection, particularly when dealing with different data sets and bioactivity prediction tasks. To further support these findings, performance metrics were also aggregated *per* type of features and algorithm (Figure S4), confirming the higher performances of models based on RDKit’s descriptors and ECFP4 fingerprints. These trends were statistically validated through Friedman’s tests and pairwise Wilcoxon signed-rank comparisons, with Bonferroni’s correction applied to control for multiple testing. Statistically significant differences were observed across aggregated performance metrics (Table S8), particularly underscoring the superior reliability of XGB and GBM models in understanding complex feature interactions with respect to ET and RF. These findings were further confirmed by statistical testing (Friedman and Wilcoxon tests, *p* < 0.001; Table S8).

### Analysis of Features and SHAP Ranges Separation for Misclassification Reduction of Compounds

Contributions of SHAP values were computed for all developed ML classifiers to investigate features’ importance. In particular, the *Tree.Explainer* method of the SHAP Python library,^50^ optimized for tree-based algorithms, was employed to calculate both *per*-molecule SHAP values and mean absolute SHAP contributions. While the analysis of SHAP values *per*-molecule enabled detailed, instance-level analyses of features’ importance on predictions, the averaged SHAP values provided a broader perspective on their rankings across the data sets, algorithms, and types of descriptors. The percentages of features with non-zero mean absolute SHAP values were assessed for all models (Figure S5). Interestingly, a subset of features in models based on ECFP4 and *custom-fragment* features exhibited SHAP values equal to zero, indicating no relevance on models’ predictions. This result was unexpected considering that RFEcv was embedded in models’ training to exclude less informative, redundant features. For ECFP4-based models, the presence of noncontributing features can be due to bit collisions, in which multiple substructures are mapped to the same bit of the fingerprints. In the case of *custom-fragment* models, this results from low fragments’ prevalence, with several fragments potentially appearing too infrequently across the data set to significantly influence models’ predictions. Notably, the proportion of null-contributing features was highest in the LNCaP data set, which is the smallest among the three cell lines evaluated, potentially reflecting its reduced chemical diversity. In contrast, models based on MACCS keys and RDKit’s descriptors consistently demonstrated meaningful contributions from all retained features. Importantly, the best-performing models based on RDKit’s descriptors (Table 2) retained only informative features, as determined through RFEcv. An analysis of cumulative contributions of SHAP values revealed that the developed classifiers required a different number of features to achieve 90% of total predictive importance (Figure 2). In particular, classifiers based on MACCS keys achieved 90% cumulative SHAP importance with approximately 20 features, highlighting their focused chemical representation; this also confirmed the compactness and limited information content of MACCS keys in the context of the studied data sets. Models based on RDKit’s molecular descriptors consistently required a higher number of features to achieve similar cumulative importance (Figure 2), reflecting their higher descriptive nature. In particular, models trained on the PC3 data set required approximately 55 to 100 RDKit descriptors, while DU-145 models required 100 to 125, and LNCaP classifiers relied on 45 to 80 descriptors to explain at least 90% of the predictions. Interestingly, models trained on ECFP4 fingerprints required an even greater number of features (*i.e.*, approximately 400) to achieve 90% cumulative SHAP value contributions. This indicates a more distributed contribution across features and may reflect the granularity of atom-centered substructural information encoded by ECFP4. Moreover, certain ECFP4 models exhibited instability, particularly when very few features were retained by RFEcv. For example, the DU-145’s ET and LNCaP’s RF ECFP4 models showed unusually limited sets of features, despite the overall requirement for broader feature inclusion in this type of fingerprints (Table S6, Figure 2). According to the performed analyses, *custom-fragments* classifiers were generally consistent between cell lines and algorithms, if compared to other feature representations and required less than 200 to attain 90% of cumulative contribution (Figure 2), confirming that only specific fragments exert a significant influence on models’ prediction performances.

**Figure 2:**
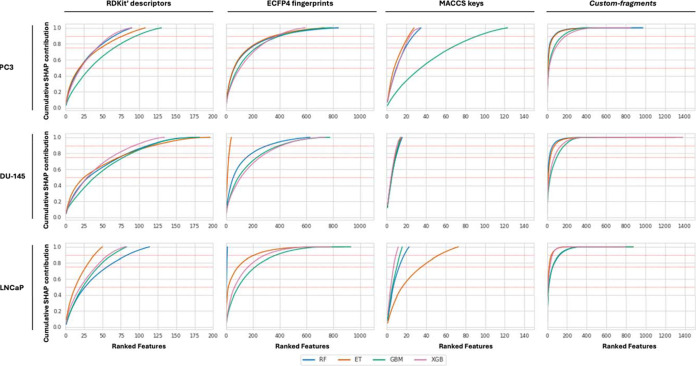
Cumulative SHAP contribution of the features remaining in each developed ML classifier after RFEcv. Features are ranked in each plot from the most (left) to the least (right) relevant ones. The red lines in the plots represent the 50%, 75%, and 90% of cumulative SHAP contribution.

These findings suggest that more efficient models based on *custom-fragments* or ECFP4 fingerprints could be trained through a two-step strategy, the first one identifying relevant features *via* SHAP analysis followed by dedicated training using the more relevant selected ones. Given that GBM models trained on RDKit’s molecular descriptors consistently achieved almost always the highest performances across all data sets (MCC_PC3_ = 0.59; F1-score _PC3_ = 0.84; MCC_DU‑145_ = 0.60; F1-score_DU‑145_ = 0.83; MCC_LNCaP_ = 0.62; F1-score_LNCaP_ = 0.88; Table 2), models based on this type of algorithm were selected for the subsequent SHAP-based analyses. In particular, our attention focused on the identification of potentially misclassified compounds, in order to improve models’ predictions performances, by exploiting the generated raw features (hereafter referred to as “RAW”) and SHAP values; this is the first study reporting the exploitation of SHAP analysis to improve models’ predictions after application. To this aim, we first evaluated and compared the “RAW” and SHAP value ranges of the active and inactive compounds in the test sets of each cell line. Notably, “RAW” and SHAP values of active and inactive test set compounds did not provide relevant differences in their ranges; to some extent, this finding is consistent with the high structural diversity highlighted in the overall data sets (Figure S1) and the test sets under evaluation (Figure S6). This suggests that a comprehensive approach based on the analysis and comparison of “RAW” and SHAP values do not allow the development of frameworks able to improve prediction performances. Therefore, further analyses of “RAW” and SHAP values were performed on more focused chemical subspaces of the data sets. To this aim, molecules of each test set were first grouped using a clustering pipeline combining Bemis-Murcko scaffolds identification, with 2D fingerprints-based similarity estimations, as detailed in the “Experimental Methods” section. This clustering strategy enabled the identification of clusters of compounds sharing significant structural similarity, which facilitates more targeted and interpretable analyses of “RAW” and SHAP value contributions within their chemical spaces. The distribution and structural diversity of the resulting clusters were systematically evaluated (Table 3).

**Table 3:** Number of Clusters Identified by Analysis of the Compounds in the PC3, DU-145, and LNCaP Data Sets^a^

Data set	Number of clusters	Number of clusters with ≥3 compounds^b^	Number of clusters with ≥5 compounds^b^
PC3	918	395 (76.6%)^c^	205 (32.4%)^c^
DU-145	447	301 (96.9%)^c^	250 (72.7%)^c^
LNCaP	152	93 (93.4%)^c^	79 (70.0%)^c^

a The number of clusters including at least 3 and 5 compounds of either active or inactive classes is also reported.

b Include only clusters containing either active and inactive ligands.

c Percentages related to the number of clusters.

The PC3 test set exhibited the highest number of clusters (*i.e.*, 918 groups), approximately doubling that of DU-145 (*i.e.*, 447 clusters) and nearly six times that of LNCaP (*i.e.*, 152 clusters). Notably, this significant difference in terms of number of clusters is consistent also with the higher number of fragments generated on PC3's chemical space compared to those of DU-145 and LNCaP data sets (Table S4). As a result, PC3 clusters, on average, contained fewer molecules, leading to sparser statistical representation within individual clusters and posing additional challenges for threshold determination. A key requirement for our range-based evaluation was the inclusion of clusters containing at least three compounds with representation of both active and inactive classes. These constituted approximately 77% of the clusters in the PC3 data set and over 93% in both the DU-145 and LNCaP models (Table 3). Increasing the minimum cluster size to five compounds (including both actives and inactives) substantially reduced the number of qualifying clusters, *i.e.*, up to 32% in the PC3 data set and approximately 73% and 70% for the DU-145 and LNCaP data sets, respectively (Table 3). This trend was observed augmenting the minimum clusters’ size, which is consistent with the fact that clustering the PC3 data set produced a significantly higher number of smaller clusters compared to DU-145 and LNCaP. These cluster statistics reinforced the critical need for adaptive, *per*-cluster thresholding and provided essential context for interpreting subsequent improvements in misclassification detection and prediction reliability. On these bases, the analysis of “RAW” and SHAP values was carried out on clusters with at least three molecules and including both active and inactive compounds. For each prostate cancer cell line classifier, a number of clusters showed separated ranges of descriptor values for active and inactive compounds (Table 4). Importantly, some features exhibited nonoverlapping value ranges between active and inactive populations within numerous clusters, highlighting their potential utility in enhancing classification reliability. Analyses of the PC3 data set revealed that approximately 50% of the descriptors presents nonoverlapping “RAW” value ranges between active and inactive compounds within the clusters.

**Table 4:** *Per*-Cluster Nonoverlapping “RAW” and SHAP Value-Ranges of Active and Inactive Compounds in the PC Test Sets

Data set	Number of RDKit’s molecular descriptors	Percentage of clusters without overlap		Number of features with ≥20% clusters without overlap	
		“RAW” (%)	SHAP (%)	“RAW”	SHAP
PC3	130	19.7	23.2	64 (49%)	114 (88%)
DU-145	180	17.5	20.0	46 (43%)	91 (85%)
LNCaP	83	18.2	18.2	25 (30%)	24 (29%)

Notably, 88% of RDKit’s descriptors displayed a clear separation between value-ranges of active and inactive compounds, as evaluated by SHAP values comparison (Table 4). A similar result was observed in the DU-145 data set, in which 85% of RDKit’s descriptors demonstrated SHAP value-ranges separation. In contrast, the LNCaP data set showed the lowest overall separability, with only around 30% of descriptors exhibiting distinct active/inactive ranges in either “RAW” or SHAP values. Across the analyzed prostate cancer test sets, most of the descriptors exhibited clear separation in both “RAW” and SHAP value-ranges between active and inactive compounds within the defined clusters (Table S9). From this, we evaluated whether misclassified and correctly classified molecules present different prevalence of “RAW” and SHAP values in the opposite class of prediction. In this phase, we considered only the top 20 features (Figures S7–S9), as they are generally considered to be the most relevant for cumulative information.^41,51,52^ Moreover, clusters in which fewer than 10% of descriptors exhibited any form of class-separating behavior were considered less informative and therefore excluded from the analyses.

Across all test sets, each compound possessed at least one descriptor with “RAW” or SHAP values that fell within ranges of the opposite class of prediction. However, misclassified compounds consistently contained a higher number of such features (Table 5), suggesting their proximity to the models’ decision boundaries and, consequently, their higher likelihood of classification uncertainty.

**Table 5:** Averaged Number of Features with Values in the Opposite Range Per Class of Prediction and Cell-Line, as Evaluated for RDKit’s Molecular Descriptors “RAW” Values and SHAP Contributions

Test set	Class	Number of compounds	Averaged number of features with RAW value in the opposite range^a^	Averaged number of features with SHAP value in the opposite range^a^
PC3	TP	1968	11.5 ± 2.7	6.4 ± 2.0
	TN	1151	14.0 ± 2.9	8.3 ± 2.3
	FP	463	22.0 ± 3.8	14.8 ± 2.9
	FN	295	25.9 ± 3.8	14.9 ± 2.8
	correctly classified	3119	12.4 ± 2.8	7.1 ± 2.1
	misclassified	758	23.5 ± 3.8	14.9 ± 2.9
DU-145	TP	1557	41.2 ± 6.1	25.4 ± 4.6
	TN	1061	29.0 ± 5.4	19.2 ± 4.2
	FP	370	55.8 ± 7.3	43.2 ± 6.1
	FN	271	44.0 ± 6.7	29.5 ± 5.2
	correctly classified	2618	36.3 ± 5.8	22.9 ± 4.4
	misclassified	641	50.8 ± 7.1	37.4 ± 5.8
LNCaP	TP	651	19.5 ± 2.5	16.3 ± 2.2
	TN	242	34.4 ± 2.5	30.8 ± 2.3
	FP	120	47.1 ± 2.6	44.8 ± 2.4
	FN	55	54.1 ± 2.6	44.5 ± 2.6
	correctly classified	893	23.5 ± 2.7	20.2 ± 2.3
	misclassified	175	49.3 ± 2.6	44.7 ± 2.5

a the average is evaluated on generated clusters.

In the LNCaP test set, which is the smallest one, misclassified compounds displayed substantially higher rates of opposite-range “RAW” values compared to correctly predicted compounds, *i.e.*, 47.1% and 54.1% for the false-positive and false-negative predictions, respectively, versus 19.5% and 34.4% for true-positive and true-negative predictions (Table 5). A comparable pattern emerged for SHAP values, with misclassified molecules again showing consistently higher percentages of opposite-range values. The greatest disparity was observed among false negatives, where misclassified molecules averaged 54 “RAW” features outside their class range, versus 24 for correctly classified compounds. The descriptors contributing most prominently to misclassifications were “EState_VSA1”, “SMR_VSA6”, “MinPartialCharge”, “Chi1”, and “PEOE_VSA3” (Table S9; misclassified: “RAW” ≈ 54.3–76.6%, “SHAP” ≈ 48.0–60.6%). These findings suggest that errors in this test set may be linked to the subtle interplay between molecular polarity and topology in LNCaP cells.

Similar trends in “RAW” and SHAP rates of opposite-range were observed for the DU-145 and PC3 models (Table 5), although the differences between misclassified and correctly classified compounds were slightly less pronounced. In the PC3 test set, correctly classified compounds had on average 12 “RAW” features within the opposite-class range, compared with 23 for misclassified compounds. A similar relationship was observed for SHAP values comparison, with averages of 7 features for correctly classified compounds and 15 for misclassified ones. Misclassified molecules showed the highest “RAW” and SHAP contributions for the descriptors “VSA_EState4”, “fr_furan”, “BalabanJ”, “fr_allylic_oxid”, and “AvgIpc” (Table S9; misclassified: “RAW” ≈ 11.6–31.9%; SHAP ≈ 10.7–12.1%). Notably, these descriptors encode a combination of electronic state indices, substructure presence (*e.g.*, furan and allylic oxidation motifs), and molecular shape complexity (*e.g.*, “BalabanJ”), suggesting that errors in prediction may arise from specific heteroaromatic or electronically rich scaffolds whose activity profiles deviate from the dominant trends in the training data. For DU-145, correctly classified compounds averaged 36 “RAW” features outside their class range compared to 51 for misclassified compounds; the SHAP-based averages were 23 and 37 features, respectively. The descriptors with the largest “RAW” and SHAP contributions to incorrect predictions were “SMR_VSA10”. “fr_bicyclic”, “NumAromaticHeterocycles”, “EState_VSA4”, and “fr_phenol_noOrthoHbond” (Table S9; misclassified: “RAW” ≈ 25.6–34.6%; SHAP ≈ 19.0–21.1%). This finding highlights the role of molecular polarizability, complex bicyclic frameworks, and phenolic functionalities in this test set, which may drive ambiguous predictions due to their variable influence on bioactivity depending on substitution patterns and local chemical environments. Collectively, these findings underscore the importance of the context-specific interpretation of molecular features. In particular, while some misclassifications are driven by structural motifs (*e.g.*, the presence of aromatic heterocycles, or hydrophobic surface area patterns) across all the test sets, others resulted to be specific *per* cell line. This variability reinforces the utility of *per*-cluster “RAW” and SHAP analyses for identifying feature-specific sources of predictive uncertainty and underscores the importance of cell-line-specific thresholding. More importantly, it suggests that some descriptors with “RAW” or SHAP values falling within the opposite-class range may be a robust indicator of potential misclassification. This approach, tailored *per* cluster and cell line, can improve both the reliability and interpretability of machine learning predictions.

### Identification of Hierarchical Quantile Thresholds for Misclassification Reduction

We next assessed whether features with “RAW” and SHAP values that fall outside the range of their assigned class could be exploited to improve models’ prediction performance. To this end, we developed a strategy to identify potentially misclassified compounds by applying *per*-cluster, value-specific thresholds determined through a hierarchical quantile approach. For each feature *M* (either the “RAW” or SHAP values), thresholds were defined as described in eqs 1 and 2 in the Experimental Methods section. Briefly, a global threshold (*T*_glob_) was obtained at different percentiles (*e.g.*, 80-th, 85-th, 90-th, or 95-th) with the highest F1-score of *M* among all correctly predicted molecules (Table S10). Then, a *per*-cluster threshold (*T*_C_) was calculated with the same percentile’s range of correctly predicted molecules within the cluster *C* (Table S10). Clusters containing fewer than three correctly classified compounds, and clusters containing molecules of only one class of prediction, were discarded, as detailed in the Experimental Methods section. For PC3 and DU-145 cell lines, *T*_glob_ and *T*_C_ thresholds were defined at 80-th percentile for “RAW” features and 85-th for SHAP values, while for LNCaP cell line best thresholds for both *T*_glob_ and *T*_C_ were defined at 85-th percentile for “RAW” features and 80-th for SHAP values (Table S10). Analyses with global and *per*-cluster thresholds defined with different percentiles of *M* among all correctly predicted molecules obtained overall poorer prediction performances (Table S10). Afterward, for each molecule *i* in cluster *C*, the number of features whose values fell within the range of the opposite prediction class in terms “RAW” or SHAP values was computed (hereafter referred to as “opposite count”). A molecule marked as potentially misclassified (hereafter referred to as “flagged”) if its “opposite count” was greater than or equal to the defined *T*_C_ thresholds. Two additional flagging rules were also derived from this framework; one approach, hereafter referred to as “RAW OR SHAP”, in which a molecule was flagged if either the “RAW” or SHAP “opposite count” exceeded the relevant threshold, maximized sensitivity and recovery of potential misclassifications. Moreover, we also derived a second approach, hereafter referred to as “RAW AND SHAP”, in which a molecule was flagged only if both “RAW” or SHAP “opposite count” exceeded the defined *T*_C_ thresholds, increasing precision at the cost of sensitivity. This latter approach ensures a higher degree of precision, lowering overall sensitivity, with populated clusters receiving stringent, data-driven thresholds, while sparse clusters fall back on the global statistic and are not overpenalized. The applicability of these thresholding strategies varied by data set (Table 6). In the LNCaP test set, over 45% of the 140 clusters met the prerequisites for defining the ″opposite class” thresholds for both “RAW” and SHAP values. In contrast, the larger and more chemically diverse DU-145 and PC3 test sets exhibited a lower proportion of applicable clusters, *i.e.*, 28.0% and 20.2%, respectively.

**Table 6:** Clusters in Each PC Best-Performing Classifier, in which Flagging Rule Thresholds Could Be Correctly Defined

Data set	Number of clusters in the test set	Number of clusters defined flagging rule thresholds^a^
LNCaP	140	64 (45.7%)
DU-145	436	122 (28.0%)
PC3	786	159 (20.2%)

a numbers within round brackets report the percentage of the clusters with respect to the total identified in the test set.

These findings suggest that data sets with smaller, more homogeneous clusters, such as the LNCaP cell line, are particularly well-suited to adaptive, cluster-specific thresholding approaches, as their structural compactness allows for more reliable local threshold estimations. In contrast, larger and more heterogeneous data sets, such as PC3, tend to rely more heavily on global feature thresholds, since their broader chemical diversity and uneven cluster composition can limit the robustness of *per*-cluster statistics. Applying this thresholding framework, we evaluated the ability of the different flagging rules to remove incorrectly and correctly classified molecules across all cell lines (Table 7).

**Table 7:** Percentages of Flagged Molecules in the Misclassified and Correctly Classified Populations (within Round Brackets), for Each PC Test Set, Based on the 4 Different Types of Flagging Rules^a^ ^,^ ^b^

Flagging rule	“RAW”	SHAP	“RAW OR SHAP”	“RAW AND SHAP”
LNCaP	48.6% (16.5%)	46.2% (13.8%)	63.6% (23.2%)	31.2% (7.1%)
PC3	19.0% (7.6%)	7.5% (4.0%)	20.7% (8.3%)	5.8% (3.2%)
DU-145	21.5% (7.9%)	21.7% (7.1%)	24.9% (10.2%)	18.3% (4.9%)

a The number of misclassified and correctly classified compounds for the LNCaP test set was 175 and 893, respectively. The numbers of misclassified and correctly classified compounds for the DU-145 test set were 641 and 2618, respectively. The numbers of misclassified and correctly classified compounds for the PC3 test set were 758 and 3119, respectively.

b Percentages were evaluated with respect to the number of misclassified and correctly classified compounds. Numbers within round brackets refer to correctly classified compounds, as identified by the flagging rules, within the correctly classified ones.

Applying the “RAW” flagging rule consistently removed substantial proportions of misclassified compounds, *i.e.*, 48% in the LNCaP test set and approximately 21% and 19% in the DU-145 and PC3 test sets, respectively, while maintaining relatively low percentages of flagged compounds among those correctly classified (Table 7). Thresholding based on “RAW” flagging therefore offers a robust and computationally efficient strategy for post-prediction screenings. Importantly, the possibility to compare the descriptors of the molecules under investigation with the ranges associated with the prediction classes in the clusters is expected to facilitate models’ interpretability, providing also insights on properties potentially optimizable in the compounds.

SHAP flagging provided results broadly similar to those of “RAW”-based rules (Table 7), with the added advantage of revealing the relative importance of individual features in driving model predictions. Combining “RAW” and SHAP flagging rules provided significantly different results, according to the type of combination. In particular, the “RAW OR SHAP” flagging rule achieved the highest sensitivity, identifying approximately 64% of misclassifications in the LNCaP test set, 25% in DU-145, and 21% in PC3, albeit at the expense of a higher proportion of flagged compounds among correctly classified predictions (Table 7). This approach offers particular value in prospective virtual screening campaigns involving large compound libraries, where false positives are common, and experimental validation resources are often limited. By maximizing sensitivity, this method can help to identify and remove compounds that show unusual patterns, either in their raw descriptor values or in the model-derived SHAP features. In contrast, the “RAW AND SHAP” rule resulted in the lowest proportion of flagged compounds in both correctly classified and misclassified populations (Table 7), reflecting its high precision but reduced sensitivity. Compared to **“**RAW OR SHAP**”**, this latter approach inevitably identifies fewer borderline cases, substantially reducing the risk of discarding potential hit compounds. This makes the “RAW AND SHAP” filtering approach particularly well-suited for focused virtual screening efforts (*e.g.*, secondary or follow-up phenotypic screenings), in which chemical diversity is narrower, data set quality is higher, and the primary aim is to preserve true positives predictions.

Finally, we assessed the performance of the proposed flagging rules in detecting potentially misclassified predictions across varying levels of prediction confidence, as estimated by using the *predict_proba* function implemented in the SciKit-Learn library. To this end, we first evaluated the distribution of misclassified and correctly classified compounds retained at different confidence thresholds (Table S11). Increasing the confidence threshold of models substantially reduced the proportion of misclassified compounds but also markedly decreased the total number of predictions assigned to a class label. For example, in the PC3 test set, increasing the threshold from 50% to 90% reduced the number of classified compounds from 3877 (19.6% misclassified) to 933 (3.6% misclassified). Similarly, in DU-145, the number decreased from 3204 (18.3% misclassified) to 768 (4.0% misclassified) and in LNCaP from 1066 (16.2% misclassified) to 599 (4.5% misclassified) (Table S11). Applying the developed flagging rules to these predictions further reduced the proportion of misclassified compounds (Figure 3). The highest increases in prediction performance were observed with the “RAW” and “RAW OR SHAP” flagging rules in the PC3 and LNCaP test sets and with the SHAP and “RAW OR SHAP” rules in DU-145 (Figure 3, Table S12).

**Figure 3:**
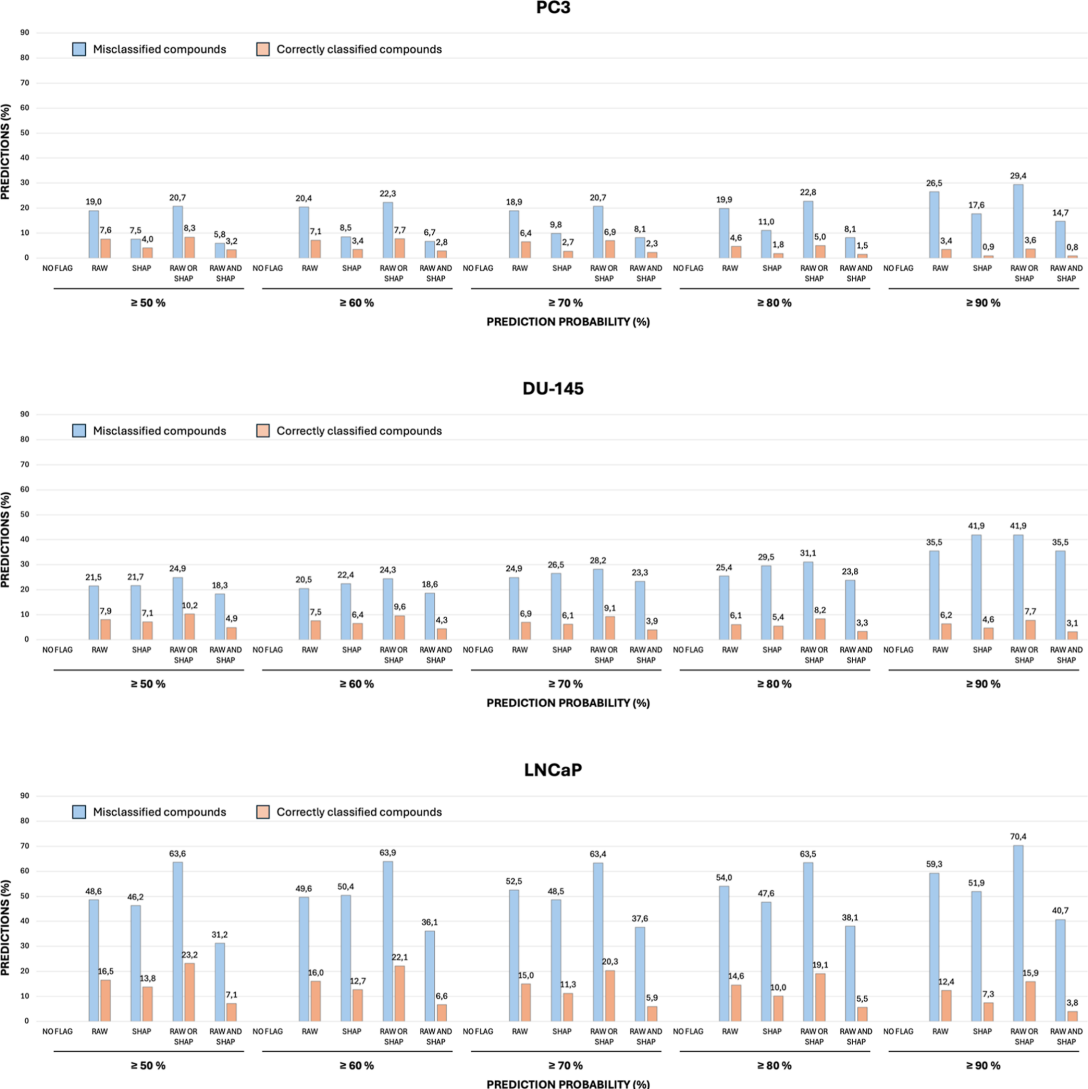
Percentages of misclassified and correctly classified compounds removed from models’ predictions, according to the proposed flagging rules. For each test set, the percentages are evaluated on populations of misclassified and correctly classified compounds predicted by the models at varying levels of confidence.

These rules consistently removed the largest fraction of misclassified compounds, although at the cost of removing higher percentages of correctly classified molecules. The flagging rules performed best when applied to predictions of the LNCaP data set. Notably, the proportion of compounds removed by the flagging rules increased with the model confidence threshold, from 50% to 90% (Figure 3, Table S12).

For example, the percentages of misclassified compounds removed by the “RAW OR SHAP” flagging rule increased from around 21% to 29%, increasing the model’s confidence (*i.e.*, *predict_proba*) threshold from 50% to 90% (Figure 3, Table S12) in the PC3 test set. These trends were even more evident when applying the “RAW OR SHAP” flagging rule in the DU-145 (removed from 24.9% to 41.9% of the misclassified compounds) and LNCaP (removed from 63.6% to 70.4% of the misclassified molecules) test sets (Figure 3, Table S12). These results, which were consistent across the different flagging approaches and test sets (Figure 3, Table S12), indicate that the reported rules are effective even in high-confidence prediction scenarios, suggesting that their application at elevated confidence thresholds can further enhance overall models’ prediction reliability. While the developed flagging rules allowed us to identify substantial percentages of misclassified compounds in models’ predictions, some challenges can be anticipated in their practical application. First, the framework developed in this study can be applied to evaluate the predictions made by ML classifiers based on tree-based algorithms. Although these methods are among the most widely used in drug design settings, the generalization of the proposed approach to other model classes (*e.g.*, deep neural networks or graph-based architectures) remains to be validated. Extending the framework to these models would require adapting SHAP computation methods and ensuring consistent interpretability across different families of models, which will need to be further addressed in future research. Second, the definition of flagging thresholds requires the test set to contain sufficiently populated and chemically homogeneous clusters, including both active and inactive compounds. This requirement may be difficult to meet in phenotypic screening campaigns, where ML classifiers are often trained on highly heterogeneous compound libraries. Nevertheless, this issue is expected to be considerably mitigated in target-related screening contexts, where the compounds in the training and test sets typically exhibit lower chemical diversity and share a well-defined mechanism of action. Future efforts should explore adaptive statistical corrections or uncertainty-aware weighting schemes to extend the framework to under-represented regions of chemical space. In addition, the computational cost associated with calculating SHAP values for large compound libraries remains non-trivial, especially in high-throughput virtual screening scenarios. Finally, the flagging rules, in particular, the “RAW OR SHAP” and “RAW AND SHAP”, involve compromises between sensitivity and precision. While the combined “RAW OR SHAP” rule achieved the highest rates of misclassification detection (up to 63% in the LNCaP data set), it also removed a greater proportion of correctly classified compounds. Conversely, the “RAW AND SHAP” rule offered a higher precision but a lower recall. Future research should therefore focus on developing more dynamic and context-aware flagging schemes able to balance these competing objectives based on the specific requirements of a given screening campaign.

Despite this, the adaptive hierarchical quantile thresholding framework represents an effective post-prediction refinement tool that not only flags potentially misclassified molecules for further review, or exclusion, but also offers significant advantages in pre-screening contexts. By enabling rapid prioritization based on descriptor-range consistency within clusters, this approach enhances both the efficiency of virtual screening workflows and the interpretability and robustness of machine learning-based predictive models.

## Conclusions

In this study, we propose a novel method that integrates SHAP and “RAW” values analysis to improve the reliability of classifiers’ predictions and reduce compounds misclassification. To this aim, we first systematically investigated how different molecular representations (*i.e.*, RDKit’s descriptors, ECFP4 fingerprints, MACCS keys, and custom-fragments) and tree-based algorithms (*i.e.*, Extra Trees, Random Forest, Gradient Boosting, and XGBoost) affect the reliability and interpretability of models predicting antiproliferative activities. Across all data sets, models built on RDKit’s molecular descriptors and ECFP4 fingerprints consistently achieved the highest predictive performance, MACCS keys yielded intermediate accuracy, and models based on custom-fragment representations displayed the lowest performance (Table 2). Among algorithms, gradient-boosting models (*i.e.*, GBM, XGB) consistently outperformed the Extra-Trees and Random Forest (Table 2).

Comprehensive SHAP analyses revealed that several features, particularly in the ECFP4 and *custom-fragment* models, contributed negligibly to predictions, even after rigorous feature elimination with RFEcv. These results highlight the added value of *post hoc* SHAP analysis as a complementary feature-pruning strategy to improve training efficiency and models’ accuracy.

Focusing on the best-performing GBM models trained on RDKit’s descriptors (Table 2), we then developed and applied an adaptive hierarchical quantile thresholding method, which integrates both raw features (herein termed “RAW”) and SHAP value contributions. The adopted *per*-cluster approach enabled the identification of a substantial fraction of misclassified compounds in models’ predictions. The “RAW” flagging rule offered a rapid and computationally efficient alternative for post-prediction triaging; this approach was able to identify a substantial portion of misclassifications (Figure 3, Table S12), potentially facilitating users in directly comparing molecular descriptors values of misclassified molecules with ranges of known active and inactive compounds. The SHAP flagging rule provided as well favorable prediction performances in identifying misclassified compounds, in particular in the DU-145 test set (Figure 3); as SHAP values represent an objective quantification of a model’s prediction among its input features, predictions made with such a flagging rule offer interpretable insights into the reasons behind resulting predictions. The highest removal rates were obtained with the combined “RAW OR SHAP” flagging rule (*i.e.*, based on the union of the “RAW” and SHAP rules). This flagging rule allowed us to remove up to 21%, 25%, and 63% of misclassified predictions in the PC3, DU-145, and LNCaP test sets, albeit at the cost of a greater loss of correctly classified compounds. In contrast, the more conservative “RAW AND SHAP” rule (*i.e.*, based on the intersection of the “RAW” and SHAP rules) delivered higher precision, preserving a greater proportion of correct predictions while reducing misclassification rates. Finally, we applied the developed flagging rules in combination with the widely used *predict_proba*-based confidence filtering approach implemented in the Python SciKit-Learn library. Increasing the confidence threshold (*e.g.*, from 50% to 90%) consistently reduced the number of misclassified predictions; however, it also substantially decreased the number of compounds with an adequate confidence prediction. Applying the flagging rules in combination with high *predict_proba* thresholds further improved the overall prediction quality, suggesting that these strategies might be employed in combination in virtual screenings. Indeed, *predict_proba* filtering is able to remove globally uncertain predictions, whereas the proposed flagging rules are demonstrated to detect misclassified compounds even when classifier confidence is high, thereby mitigating a key limitation of probability-based screenings. By leveraging adaptive, cluster-specific thresholds on “RAW” and SHAP value ranges, the flagging approach developed here serves as an effective post-prediction refinement strategy, either as a standalone method or in combination with complementary techniques, such as *predict_proba*-based filtering. This integrated use can facilitate the identification of potentially misclassified compounds in large-scale virtual screening campaigns while simultaneously enhancing both the interpretability and the robustness of the applied ML classifiers.

## Experimental Methods

### Curation of Data Sets of Compounds for Selected PC Cell Lines

A series of ML classifiers was first developed on data sets of compounds with antiproliferative activity records against PC3, DU-145, and LNCaP prostate cancer cell lines. Compound structures and corresponding antiproliferative activity data for these cell lines were first retrieved from the ChEMBL database (https://www.ebi.ac.uk/chembl/; release 29; accessed on: 2021-11-21),^53^ following our interest in prostate cancer drug discovery.^6,54,55^ Then, the data sets were curated and filtered in order to retain molecules with (i) “*Standard Type*” equal to IC_50_, GI_50_, EC_50_, ED_50_; (ii) “*Standard Relation*” equal to “ = , >, <”; (iii) “*Standard Unit*” equal to “nM”; (iv) “*Target type*” equal to “CELL-LINE”; (v) “*Target Organism*” equal to “Human”; (vi) “*Assay Type*” equal to “F” (*i.e.*, functional assay type); (vii) “*Assay Organism*” equal to “*Homo Sapiens*”; (viii) “*BAO Label*” equal to “Cell-based format”; and (ix) “*Assay description*” that clearly reported assays performed at 24, 48, 72, or 96 h timetables, and with MTT, SRB, MTS, or CCK8 assay methods. For each compound, activity records were deduplicated, retaining the annotation closest to the weighted average of all reported values. Compounds with % inhibition lower than 50%, resulting from antiproliferative tests at 10 μM concentration, were also included at this stage to improve the number of “inactives”, therefore obtaining more class-balanced data sets. Collectively, the adopted screening protocol allowed to obtain 10,505 compounds for PC3, 9004 for DU-145, and 3002 for LNCaP (see Supporting File 1). Afterward, filtered molecules were labeled as “active” or “inactive” according to an activity threshold equal to 10 μM (*i.e.*, molecules with activity values higher than the defined threshold were considered inactives, while others were considered as actives). ML classifiers were developed based on different types of features (see below) in order to investigate the effects of multiple chemical representations on the prediction performances of models and to identify potential advantages and limitations of different levels of chemical abstraction. To this aim, the chemical structure of each compound was standardized through canonical SMILES generation, followed by removal of salts and formal charges adjustment using the RDKit’s Python library (version 2022.03.1). Then, 220 RDKit’s molecular descriptors were generated with default settings. Besides, MACCS Keys and ECFP4 fingerprints (1024 bits-length) were computed employing *in-house*-developed scripts implemented with the RDKit Python library and stored as separated data sets. Twelve MACCS keys were consistently removed across all three cell line data sets, as they encode for patterns less commonly present in drug-like compounds and complex metal-containing substructures (Table S1). The impact of bit collisions in ECFP4 fingerprints was assessed and analyzed in relation to the size of each data set. Random samples of compounds were extracted at increasing percentages (*i.e.*, 20%, 40%, 60%, 80%, 100%) of the overall data set size, keeping into account the active/inactive balance, and their average bit collision and standard deviation were evaluated at each percentage. Statistical assessments using Pearson’s and Spearman’s correlation coefficients were conducted with the SciPy Python library (version 1.7.3)^56^ to confirm the relationship between bit collisions frequency and data set size. Besides, recursive molecular fragmentation was performed using established methods, namely, Bemis-Murcko,^49^ RECAP,^48^ and BRICS,^57^ and rings detachment to derive structural features specific to the PC3, DU-145, and LNCaP data sets. These features, referred to as “*custom-fragments*”, were generated by decomposing parent compounds into substructures. The fragmentation protocol preserved explicit hydrogen atoms, atomic connectivity, and stereochemical information. Resulting fragments were encoded as SMARTS-based patterns, and duplicates were removed obtaining 157,472 unique fragments for PC3, 138,804 for DU-145, and 62,595 for LNCaP. Afterward, fragments were ranked within each data set based on their prevalence in the parent compounds. To ensure statistical relevance and reduce noise, fragments occurring in fewer than 20 molecules per data set were excluded, resulting in final fragment sets including 1105 for PC3, 1423 for DU-145, and 1043 for LNCaP. These curated fragment sets were then used to generate “*custom-fragment*” fingerprints for each compound, using in-house scripts developed with the RDKit Python library. For each cell line data set, compounds were partitioned into training and test sets following a 70:30 ratio. A custom splitting strategy was employed to maintain a balanced representation across activity classes, ensuring that the chemical diversity within each class was preserved in both subsets.

### Training of ML Classifiers, SHAP Evaluations, and Statistical Analyses

Four different types of tree-based classification algorithms, including ET, RF, GBM, and XGB, were used to train ML classifiers for antiproliferative activity prediction on PC3, DU-145, and LNCaP cells. The ET, RF, and GBM classifiers were implemented using the SciKit-Learn library (version 1.3.2),^58^ while XGB models were constructed using the XGBoost Python library (version 1.1.2).^37^ ML classifiers were developed with a procedure including: (i) two rounds of hyperparameters (HP) optimization with a GridSearch algorithm; (ii) elimination of redundant features by Recursive Feature Elimination; and (iii) a stratified 10-fold cross-validation (CV) to more efficiently exploit all the available data set information. A total of 3 (cell lines) X 4 feature data sets (MACCS, ECFP4, RDKit, *custom-fragments*) X 4 ML algorithms (ET, RF, GBM, and XGB) = 48 ML classifiers were developed in this study, whose performance was assessed through Accuracy, Precision, Recall, Matthews Correlation Coefficient, F1-score, and Area Under the Receiver Operating Characteristic Curve (ROC-AUC) metrics; ROC-AUC was also calculated for the training set’s CV folds. The *predict_proba* function implemented in the SciKit-Learn library was applied to assess the class assignment confidence. SHapley Additive exPlanations values were computed with the *Tree.Explainer* module from the SHAP library (version 0.41.0)^59^ to assess the contribution of individual features to model predictions. Mean absolute SHAP values were computed across all classifiers, enabling features ranking and identifying common high-impact descriptors across models, cell lines, feature sets, and algorithms. This analysis also revealed instances where features retained through RFE did not contribute relevantly to model’s predictions. Statistical significance among classifiers was evaluated by using metrics collected from CV folds. Both individual (*all-against-all*) and aggregated comparisons (by algorithm or feature type) were performed. Statistical differences were evaluated *via* Friedman’s tests for non-parametric repeated measures, followed, when appropriate, by Wilcoxon signed-rank tests with Bonferroni correction to adjust for multiple comparisons. Effect sizes were also computed. All statistical analysis were performed using NumPy (version 1.22.3),^60^ Pandas (version 1.4.1),^61^ SciPy (version 1.7.3),^56^ and Matplotlib (version 3.5.1)^62^ for data visualization.

### RDKit’s Molecular Descriptors and SHAP Features Evaluation and Threshold Rules Definition for Misclassification Analysis

For each PC data set, compounds in the test set were clustered based on the similarity of their Bemis-Murcko scaffolds. Molecular similarity was computed using Extended-Connectivity Fingerprints (ECFP4; radius = 2, 1024 bits), with the Tanimoto index^63^ employed as the similarity metric. Similarity scores were subsequently transformed to distance values. Then, Uniform Manifold Approximation and Projection (UMAP)^64^ was employed to reduce the dimensionality of the resulting high-dimensional scaffold distance matrix suitable for clustering. The reduced representations were then subjected to density-based clustering using HDBSCAN,^65^ which extracts clusters based on density variations and automatically identifies outliers as noise. Optimal UMAP and HDBSCAN parameters were selected through Silhouette score maximization to ensure robust cluster separation. Singletons (*i.e.*, non-clustered compounds) were reassigned to the nearest cluster by calculating their average similarity to cluster members, by using both MACCS and ECFP4 fingerprints. Within each resulting cluster, raw feature (“RAW”) values and corresponding SHAP values were analyzed using an “opposite-range” logic, by comparing them to the activity-class-specific min–max value ranges defined in the test set of each PC cell line. For each test set molecule and feature, the number of instances where “RAW” or SHAP values fell within the range characteristic of the opposite class was recorded. Molecules exceeding predefined thresholds for these opposite-class counts were flagged as potentially misclassified predictions. Threshold rules were established using a hierarchical quantile (*T*_C_(*M*)) methodology

1 $$T_{global}\left(M\right)={quantile}_{p}\left(M_{correct}\right)$$

2 $$T_{C}\left(M\right)={quantile}_{p}\left(M_{correct\;in\;C}\right),,\text{if}\;\left|C\right|\geq 3$$

where *M* represents the count of “RAW” and SHAP values in the opposite-ranges and *M*_correct_ to the count of raw features and corresponding SHAP values in the opposite ranges only in correctly predicted molecules. The optimal percentile *p* is the chosen one between 80-th, 85-th, 90-th, and 95-th percentiles, through 5-fold cross-validation to maximize the F1-score, commonly resulting in the selection of the 80-th and 85-th percentiles. |*C*| denotes the number of correctly predicted compounds in each cluster evaluated.

Four distinct flagging rules were applied to identify the potential critical predictions: (i) “RAW” flagging compounds whose raw feature descriptor opposite-range count is equal to or higher than max T_C_(RAW); (ii) SHAP flagging compounds, whose SHAP value opposite-range count is equal to or higher than max *T*_C_(SHAP); (iii) “RAW OR SHAP”: union of compounds flagged by either the “RAW” or SHAP criteria; and (iv) “RAW AND SHAP”: intersection of compounds flagged by both “RAW” and SHAP criteria. The performance of these filtering criteria was assessed by analyzing the proportions of misclassified and correctly classified compounds removed under each rule for the PC3, DU-145, and LNCaP test sets. This evaluation provided insights into the trade-off between sensitivity (*i.e.*, the removal of true misclassifications) and specificity (*i.e.*, preservation of correct predictions). Finally, results of the developed flagging criteria were evaluated on compounds whose class prediction showed different levels of confidence (*i.e.*, 50%, 60%, 70%, 80%, and 90%), as evaluated with the predict_proba function implemented in the SciKit-Learn library. All analyses were conducted using custom Python scripts developed *in-house*.

## Supplementary Material





## Data Availability

PC3, DU-145, and LNCaP curated data sets underlying this study are available in the Supporting File 1.

## Notes

RDKit’s molecular descriptors categorized according to the type of information they provide (Table S1); MACCS keys removed across PC3, DU-145, and LNCaP cell lines (Table S2); MACCS keys commonly present in compounds of the PC3, DU-145, or LNCaP data sets (Table S3); Number of fragments obtained from decomposition of the data sets (Table S4); Molecular fragments commonly present in compounds of the PC3, DU-145, or LNCaP data sets, as evaluated with the custom-fragment approach (Table S5); Hyperparameters configurations and number of features retained for each ML model trained in the PC3, DU-145, and LNCaP data sets (Table S6); RDKit’s descriptors commonly retained by ML models trained on PC3, DU-145, and LNCaP data sets after RFEcv (Table S7); Statistical comparisons of significative differences between aggregated training cross-validation-fold performance metrics (Table S8); Selected top RDKit’s molecular descriptors by “RAW” and SHAP values most frequently emerging from misclassified compounds of each PC test set (Table S9); Overall prediction performances evaluated for the “RAW” and SHAP flagging rules using different quantile global (Panel A) and per-cluster (Panel B) thresholds for misclassification reduction (Table S10); Numbers and percentages of misclassified and correctly classified compounds removed by models’ predictions, at different levels of confidence (Table S11); Percentages of misclassified and correctly classified compounds removed from models’ predictions according to the proposed flagging rules, at different levels of models’ confidence, for each test set (Table S12); Distribution of the compounds in the PC3, DU-145, and LNCaP data sets, according to MACCS and ECFP4 fingerprints-based similarity estimations (Figure S1); Pie charts displaying active and inactive compounds in the PC3, DU-145, and LNCaP data sets (Figure S2); Comparison of training set cross-validation fold values of performance metrics (Figure S3); Averaged values of all performance metrics, aggregated per type of features (blue columns) or algorithm (colored columns) (Figure S4); Percentage of features with a positive (blue columns) or null (orange columns) cumulative SHAP value contribution as evaluated for developed ML classifiers (Figure S5); Distribution of the compounds in the PC3, DU-145. and LNCaP test sets, according to MACCS and ECFP4 fingerprints-based similarity estimations (Figure S6); Percentage of molecules in the PC3 data set with “RAW” or SHAP values in the ranges of the opposite class of prediction, for the misclassified and the correctly classified molecules populations, considering the top 20 contributing features by mean SHAP values (Figure S7); Percentage of molecules in the DU-145 data set with “RAW” or SHAP values in the ranges of the opposite class of prediction, for the misclassified and the correctly classified molecules populations, considering the top20 contributing features by mean SHAP values (Figure S8); Percentage of molecules in the LNCaP data set with “RAW” or SHAP values in the ranges of the opposite class of prediction, for the misclassified and the correctly classified molecules populations, considering the top20 contributing features by mean SHAP values (Figure S9) (PDF)
PC3, DU-145, and LNCaP data sets (ZIP)

## Abbreviations

ROC-AUC: area under the receiver operating characteristic curve
CV: cross-validation
ET: Extra Tree
FN: false negative
FP: false positives
LB: ligand-based
ML: machine learning
MCC: Matthews correlation coefficient
PC: prostate cancer
TN: true negatives
TP: true positives
RFE: recursive feature elimination
RFECV: recursive feature elimination with cross-validation
SHAP: SHapley Additive Explanations
UMAP: uniform manifold approximation and projection
HDBSCAN: hierarchical density-based spatial clustering of applications with noise
QED: quantitative estimation of drug-likeness
ET: extra trees
RF: random forest
GBM: gradient boosting machines
XGB: XGBoost
HP: hyperparameters
CV: cross-validation
ECFP4: extended-connectivity fingerprints
