# Supplementary material for: Improving Machine Learning Classification Predictions through SHAP and Features Analysis Interpretation
Source: J Chem Inf Model. 2025 Oct 20;65(21):11716–32. doi: 10.1021/acs.jcim.5c02015 (PMC12606625; doi:10.1021/acs.jcim.5c02015)

Improving machine learning classification predictions through SHAP and features analysis interpretation

*Leonardo Bernal, [1,2] Giulio Rastelli, [1]Luca Pinzi[1] **

[1] Department of Life Sciences, University of Modena and Reggio Emilia, Via Giuseppe Campi 103, 41125 Modena, Italy.

[2] Clinical and Experimental Medicine PhD Program, University of Modena and Reggio Emilia, Modena, Italy.

*** Correspondence to**: Luca Pinzi, Department of Life Sciences, University of Modena and Reggio Emilia, Via Giuseppe Campi 103, 41125 Modena, Italy. Tel +39 059 2058625, Email: luca.pinzi@unimore.it

Table of contents

Tables

Table S1 3
Table S2 5
Table S3 6
Table S4 12
Table S5 13
Table S6 18
Table S7 20
Table S8 26
Table S9 27
Table S10 29
Table S11 30
Table S11 31

Figures

Figure S1 34
Figure S2 35
Figure S3 36
Figure S4 37
Figure S5 38
Figure S6 39
Figure S7 40
Figure S8 41
Figure S9 42

Table S1. RDKit's molecular descriptors categorized according to the type of information they provide. In particular, the "electronic and charge descriptors" category includes properties as maximum electro-topological state indexes, which locate the atom with highest combined electronegativity and connectivity. The "constitutional counts" category includes a series of descriptors summarizing molecular composition. "Topological descriptors" include molecular features related to graph-theoretic and topological indices evaluated on molecules. "3D Geometric / Shape Descriptors" reflect properties that can be evaluate on the 3D molecular structure of the compounds, including radius of gyration, sphericity and principal moments of inertia, some of them capturing conformational spread and anisotropy that can modulate steric complementarity. "BCUT descriptors" integrate atomic polarizability and topology into principal-component. RDKit's solvent-accessible surface-area descriptors (i.e., "Surface Area & VSA (MOE-type) Descriptors" category), allow to dissect the molecular surface into regions of differing polarity or refractivity, guiding predictions of solvation and membrane partitioning. "Fingerprint-density" descriptors report on the overall richness of structural motifs, thus serving as a proxy for molecular complexity. Finally, molecular descriptors, which can represent drug-likeness scores (*i.e.*, the "Drug-likeness & Physicochemical Descriptors" category), allow to approximate oral bioavailability and target-likeness.

Category	Family of descriptors	Type of information provided	Name of descriptor
ELECTRONIC / CHARGE DESCRIPTORS	EState descriptors	They capture electron distribution based on adjacency and electronegativity.	MaxAbsEStateIndex; MaxEStateIndex; MinAbsEStateIndex; MinEStateIndex
	Partial charge descriptors	Statistics of partial charges for each atom	MaxPartialCharge; MinPartialCharge; MaxAbsPartialCharge; MinAbsPartialCharge
	EState VSA descriptors	Sum of approximate Van der Waals's surface areas by EState ranges.	EState_VSA1; EState_VSA2; EState_VSA3; EState_VSA4; EState_VSA5; EState_VSA6; EState_VSA7; EState_VSA8; EState_VSA9; EState_VSA10; EState_VSA11
	VSA EState descriptors	Surface area descriptors to average EState values.	VSA_EState1; VSA_EState2; _EState3; VSA_EState4; VSA_EState5; VSA_EState6; VSA_EState7; VSA_EState8; VSA_EState9; VSA_EState10
CONSTITUTIONAL COUNTS DESCRIPTORS	Basic molecular properties	Simple sums or molecule-level properties	MolWt; HeavyAtomMolWt; ExactMolWt; NumValenceElectrons;NumRadicalElectrons
	Atom and ring counts	Counts of atoms, ring systems, and related structural features.	HeavyAtomCount; FractionCSP3; NHOHCount; NOCount; NumHAcceptors; NumHDonors; NumHeteroatoms; NumRotatableBonds; NumAliphaticCarbocycles; NumAliphaticHeterocycles; NumAliphaticRings; NumAromaticCarbocycles; NumAromaticHeterocycles; NumAromaticRings; NumSaturatedCarbocycles; NumSaturatedHeterocycles; NumSaturatedRings; RingCount
TOPOLOGICAL DESCRIPTORS	Chi (Kier–Hall) indices	Graph-theoretic indices for molecular branching and shape.	Chi0; Chi0n; Chi0v; Chi1; Chi1n; Chi1v; Chi2n; Chi2v; Chi3n; Chi3v; Chi4n; Chi4v
	Kappa (shape) Indices	Kappa shape indices for molecular geometry from 2D connectivity.	Kappa1; Kappa2; Kappa3
	Hall–Kier Alpha	Adjustment factor used in Kier–Hall topological indices.	HallKierAlpha
	Balaban's J	Topological shape descriptor based on distances in the graph.	BalabanJ
	Bertz Complexity	Molecular complexity measure derived from the graph.	BertzCT
	Information Content	Average information content index (Ipc).	AvgIpc
BCUT (2D) DESCRIPTORS	BCUT2D	Eigenvalue-based descriptors	BCUT2D_MWHI; BCUT2D_MWLOW; BCUT2D_CHGHI; BCUT2D_CHGLO; BCUT2D_LOGPHI; BCUT2D_LOGPLOW; BCUT2D_MRHI; BCUT2D_MRLOW
3D GEOMETRIC / SHAPE DESCRIPTORS	3D Shape / Geometry	Descriptors from 3D coordinates	3d_Asphericity; 3d_Eccentricity; 3d_InertialShapeFactor; 3d_NPR1; 3d_NPR2; 3d_PBF; 3d_PMI1; 3d_PMI2; 3d_PMI3; 3d_RadiusOfGyration; 3d_SpherocityIndex
SURFACE AREA & VSA (MOE-TYPE) DESCRIPTORS	Labute ASA	Approximate solvent-accessible surface area by Labute's method.	LabuteASA
	PEOE_VSA	Van der Waals's surface areas binned by partial charges	PEOE_VSA1; PEOE_VSA2; PEOE_VSA3; PEOE_VSA4; PEOE_VSA5; PEOE_VSA6; PEOE_VSA7; PEOE_VSA8; PEOE_VSA9; PEOE_VSA10; PEOE_VSA11; PEOE_VSA12; PEOE_VSA13; PEOE_VSA14

	SMR_VSA	Van der Waals's surface areas binned by atomic contributions to mol. refractivity	SMR_VSA1; SMR_VSA2; SMR_VSA3; SMR_VSA4; SMR_VSA5; SMR_VSA6; SMR_VSA7; SMR_VSA8; SMR_VSA9; _VSA10
	SlogP_VSA	Van der Waals's surface areas binned by atomic contributions to logP	SlogP_VSA1; SlogP_VSA2; _VSA3; SlogP_VSA4; SlogP_VSA5; SlogP_VSA6; SlogP_VSA7; SlogP_VSA8; SlogP_VSA9; SlogP_VSA10; SlogP_VSA11; SlogP_VSA12
FINGERPRINT DENSITY DESCRIPTORS	Morgan Fingerprint Density	Morgan circular fingerprint counts normalized by number of atoms.	FpDensityMorgan1; FpDensityMorgan2; FpDensityMorgan3
DRUG-LIKENESS & PHYSICOCHEMICAL DESCRIPTORS	General Drug-likeness Descriptors	Descriptors capturing drug-likeness, synthetic accessibility (SPS), polar surface area.	qed; SPS; TPSA; MolLogP; MolMR
CONSTITUTIONAL DESCRIPTORS	Functional Group / Substructure	Binary or integer counts for the presence of specific functional groups and substructures	fr_Al_COO; fr_Al_OH; fr_Al_OH_noTert; fr_ArN; fr_Ar_COO; fr_Ar_N; fr_Ar_NH; fr_Ar_OH; fr_COO; fr_COO2; fr_C_O; fr_C_O_noCOO; fr_C_S; fr_HOCCN; fr_Imine; fr_NH0; fr_NH1; fr_NH2; fr_N_O; fr_Ndealkylation1; fr_Ndealkylation2; fr_Nhpyrrole; fr_SH; fr_aldehyde; fr_alkyl_carbamate; fr_alkyl_halide; fr_allylic_oxid; fr_amide; fr_amidine; fr_aniline; fr_aryl_methyl; fr_azide; fr_azo; fr_barbitur; fr_benzene; fr_benzodiazepine; fr_bicyclic; fr_diazo; fr_dihydropyridine; fr_epoxide; fr_ester; fr_ether; fr_furan; fr_guanido; fr_halogen; fr_hdrzine; fr_hdrzone; fr_imidazole; fr_imide; fr_isocyan; fr_isothiocyan; fr_ketone; fr_ketone_Topliss; fr_lactam; fr_lactone; fr_methoxy; fr_morpholine; fr_nitrile; fr_nitro; fr_nitro_arom; fr_nitro_arom_nonortho; fr_nitroso; fr_oxazole; fr_oxime; fr_para_hydroxylation; fr_phenol; fr_phenol_noOrthoHbond; fr_phos_acid; fr_phos_ester; fr_piperdine; fr_piperzine; fr_priamide; fr_prisulfonamd; fr_pyridine; fr_quatN; fr_sulfide; fr_sulfonamd; fr_sulfone; fr_term_acetylene; fr_tetrazole; fr_thiazole; fr_thiocyan; fr_thiophene; fr_unbrch_alkane; fr_urea

Table S2. MACCS keys removed across PC3, DU-145 and LNCaP cell lines. A brief description of their pattern representation is also provided.

MACCS'S KEYS ID*	PATTERN	DESCRIPTION
1	'?'	Presence of Isotopes
2	Lr, Rf	Presence of Lr or Rf
3	Ge, As, Se, Sn, Sb, Te, Tl, Pb, Bi	Presence of metals or heavy post-transition elements
4	Ac, Th, Pa, U, Np, Pu, Am, Cm, Bk, Cf, Es, Fm, Md, No, Lr	Presence of actinide elements
5	Sc, Ti, Y, Zr, Hf	Presence of Group 3/4 transition metals
6	La, Ce, Pr, Nd, Pm, Sm, Eu, Gd, Tb, Dy, Ho, Er, Tm, Yb, Lu	Presence of lanthanides
7	V, Cr, Mn, Nb, Mo, Tc, Ta, W, Re	Presence of Group 5–7 transition metals
9	Fe, Co, Ni, Ru, Rh, Pd, Os, Ir, Pt	Presence of late transition metals
10	Be, Mg, Ca, Sr, Ba, Ra	Group IIA
12	Cu, Zn, Ag, Cd, Au, Hg	Group IB, IIB
35	Li, Na, K, Rb, Cs, Fr	Group IA
166	NaN	No direct chemical interpretation / Bit placeholder

Note: *as defined by in RDKit (https://github.com/rdkit/rdkit-orig/blob/master/rdkit/Chem/MACCSkeys.py)

Table S3. MACCS keys commonly present in compounds of the PC3, DU-145 or LNCaP datasets.

MACCS'S Keys ID	SMARTS PATTERNS	PC3			DU-145			LNCaP		
		N° Total	N° Actives	N° Inactives	N° Total	N° Actives	N° Inactives	N° Total	N° Actives	N° Inactives
8	[!#6;!#1]1~*~*~*~1	62	53	9	34	30	4	16	16	0
11	*1~*~*~*~1	71	58	13	43	33	10	36	34	2
13	[#8]~[#7](~[#6])~[#6]	126	71	55	108	78	30	2	2	0
14	[#16]-[#16]	49	32	17	27	20	7	18	12	6
15	[#8]~[#6](~[#8])~[#8]	6	3	3	2	0	2	5	2	3
16	[!#6;!#1]1~*~*~1	171	124	47	123	86	37	24	20	4
17	[#6]#[#6]	102	58	44	122	82	40	57	43	14
18	[#5,#13,#31,#49,#81]	22	16	6	15	14	1	2	1	1
19	*1~*~*~*~*~*~*~1	717	504	213	421	208	213	194	184	10
20	[#14]	13	8	5	16	8	8	2	2	0
21	[#6]=[#6](~[!#6;!#1])~[!#6;!#1]	48	26	22	9	6	3	10	9	1
22	*1~*~*~1	346	204	142	236	151	85	55	42	13
23	[#7]~[#6](~[#8])~[#8]	290	175	115	121	77	44	103	88	15
24	[#7]-[#8]	1241	773	468	866	466	400	205	97	108
25	[#7]~[#6](~[#7])~[#7]	608	382	226	448	246	202	202	122	80
26	[#6]=;@[#6](@*)@*	795	424	371	400	214	186	247	194	53
27	[I]	65	34	31	66	30	36	12	8	4
28	[!#6;!#1]~[CH2]~[!#6;!#1]	381	240	141	599	406	193	39	23	16
29	[#15]	105	47	58	75	44	31	14	6	8
30	[#6]~[!#6;!#1](~[#6])(~[#6])~*	114	104	10	22	11	11	3	3	0
31	[!#6;!#1]~[F,Cl,Br,I]	4	4	0	109	12	97	109	31	78
32	[#6]~[#16]~[#7]	560	362	198	402	223	179	94	51	43
33	[#7]~[#16]	582	380	202	522	314	208	103	58	45
34	[CH2]=*	536	282	254	343	178	165	67	42	25

36	[#16R]	1287	783	504	1054	534	520	365	267	98
37	[#7]~[#6](~[#8])~[#7]	772	523	249	325	172	153	263	246	17
38	[#7]~[#6](~[#6])~[#7]	2453	1640	813	1725	1000	725	751	611	140
39	[#8]~[#16](~[#8])~[#8]	34	14	20	145	100	45	28	19	9
40	[#16]-[#8]	46	20	26	172	118	54	35	21	14
41	[#6]#[#7]	307	165	142	500	193	307	265	152	113
42	F	1499	854	645	1533	773	760	697	388	309
43	[!#6;!#1;!H0]~*~[!#6;!#1;!H0]	751	509	242	376	166	210	331	251	80
44	[!#6;!#1;!H0]~*~*~*~[!#6;!#1;!H0]	138	89	49	52	34	18	2	1	1
45	[#6]=[#6]~[#7]	329	168	161	423	311	112	217	197	20
46	Br	572	283	289	575	281	294	99	70	29
47	[#16]~*~[#7]	1477	809	668	931	425	506	414	257	157
48	[#8]~[!#6;!#1](~[#8])(~[#8])	116	42	74	207	137	70	43	28	15
49	[!+0]	743	451	292	636	368	268	214	104	110
50	[#6]=[#6](~[#6])~[#6]	2240	1271	969	1385	723	662	537	402	135
51	[#6]~[#16]~[#8]	811	533	278	829	429	400	191	89	102
52	[#7]~[#7]	1758	1014	744	1328	560	768	376	291	85
53	[!#6;!#1;!H0]~*~*~[!#6;!#1;!H0]	1356	844	512	1105	750	355	322	268	54
54	[!#6;!#1;!H0]~*~*~[!#6;!#1;!H0]	1343	831	512	1158	715	443	713	434	279
55	[#8]~[#16]~[#8]	818	536	282	899	485	414	205	103	102
56	[#8]~[#7](~[#8])~[#6]	424	229	195	502	261	241	171	71	100
57	[#8R]	3445	2154	1291	2975	1735	1240	1128	885	243
58	[!#6;!#1]~[#16]~[!#6;!#1]	838	552	286	1008	510	498	287	128	159
59	[#16]!:*:*	1043	595	448	932	430	502	320	143	177
60	[#16]=[#8]	817	536	281	899	485	414	204	103	101
61	*~[#16](~*)~*	828	542	286	1007	514	493	290	129	161
62	*@*!@*@*	4265	2566	1699	3690	2025	1665	1445	1034	411
63	[#7]=[#8]	389	195	194	509	267	242	176	75	101

64	*@*!@[#16]	1097	635	462	1045	447	598	409	188	221
65	c:n	5733	3593	2140	4687	2891	1796	1308	984	324
66	[#6]~[#6](~[#6])(~[#6])~*	2491	1447	1044	1779	914	865	999	662	337
67	[!#6;!#1]~[#16]	907	590	317	1070	553	517	330	144	186
68	[!#6;!#1;!H0]~[!#6;!#1;!H0]	563	423	140	121	89	32	15	11	4
69	[!#6;!#1]~[!#6;!#1;!H0]	1538	1021	517	921	506	415	151	100	51
70	[!#6;!#1]~[#7]~[!#6;!#1]	950	532	418	961	487	474	218	96	122
71	[#7]~[#8]	1341	821	520	1048	587	461	282	147	135
72	[#8]~*~*~[#8]	4264	2750	1514	3374	2072	1302	1233	812	421
73	[#16]=*	1122	677	445	1165	567	598	345	177	168
74	[CH3]~*~[CH3]	2227	1322	905	1343	763	580	680	508	172
75	*!@[#7]@*	4469	2939	1530	3338	1974	1364	1315	994	321
76	[#6]=[#6](~*)~*	2585	1444	1141	2123	1119	1004	733	568	165
77	[#7]~*~[#7]	4100	2531	1569	2906	1557	1349	1101	800	301
78	[#6]=[#7]	1067	622	445	609	284	325	118	50	68
79	[#7]~*~*~[#7]	4391	2729	1662	3456	1945	1511	1377	998	379
80	[#7]~*~*~*~[#7]	4257	2594	1663	3071	1724	1347	834	649	185
81	[#16]~*(~*)~*	2374	1380	994	2256	1037	1219	800	471	329
82	*~[CH2]~[!#6;!#1;!H0]	2109	1325	784	1689	1115	574	688	524	164
83	[!#6;!#1]1~*~*~*~*~1	6158	3785	2373	5007	2820	2187	1650	1243	407
84	[NH2]	969	605	364	945	657	288	428	319	109
85	[#6]~[#7](~[#6])~[#6]	4561	3000	1561	3549	2227	1322	1344	1000	344
86	[C;H2,H3][!#6;!#1][C;H2,H3]	2973	2040	933	1861	1073	788	837	642	195
87	[F,Cl,Br,I]!@*@*	2702	1498	1204	2485	1270	1215	527	351	176
88	[#16]	2572	1509	1063	2631	1293	1338	851	506	345
89	[#8]~*~*~*~[#8]	4142	2567	1575	3828	2158	1670	1254	847	407
90	[$([!#6;!#1;!H0]~*~*~[CH2]~*),$([!#6;!#1;!H0;R]1@[R]@[R]@[CH2;R]1)]	3516	2209	1307	2665	1575	1090	1170	782	388
91	[$([!#6;!#1;!H0]~*~*~*~[CH2]~*),$([!#6;!#1;!H0;R]1@[R]@[R]@[R]@[CH2;R]1)]	3407	2085	1322	2530	1554	976	865	645	220

92	[#8]~[#6](~[#7])~[#6]	4535	2758	1777	3601	2019	1582	1246	769	477
93	[!#6;!#1]~[CH3]	4734	3045	1689	4141	2498	1643	1255	921	334
94	[!#6;!#1]~[#7]	3346	2026	1320	2674	1349	1325	724	479	245
95	[#7]~*~*~[#8]	4023	2469	1554	3388	1882	1506	1476	1001	475
96	*1~*~*~*~*~1	6693	4029	2664	5806	3383	2423	1784	1352	432
97	[#7]~*~*~*~[#8]	4957	2937	2020	4100	2444	1656	996	702	294
98	[!#6;!#1]1~*~*~*~*~*~1	5485	3456	2029	4577	2852	1725	1387	1021	366
99	[#6]=[#6]	4002	2387	1615	3182	1827	1355	1060	813	247
100	*~[CH2]~[#7]	4792	3142	1650	3509	2146	1363	1396	1032	364
101	[$([R]@1@[R]@[R]@[R]@[R]@[R]@[R]@[R]1),$([R]@1@[R]@[R]@[R]@[R]@[R]@[R]@[R]@[R]1)]	7203	4322	2881	5857	3372	2485	1470	1049	421
102	[!#6;!#1]~[#8]	2181	1347	834	1992	1136	856	476	250	226
103	Cl	1482	848	634	1492	744	748	247	169	78
104	[!#6;!#1;!H0]~*~[CH2]~*	3303	2099	1204	2456	1453	1003	1171	772	399
105	*@*(@*)@*	7320	4415	2905	5950	3410	2540	1643	1213	430
106	[!#6;!#1]~*(~[!#6;!#1])~[!#6;!#1]	3230	1897	1333	2449	1197	1252	1148	704	444
107	[F,Cl,Br,I]~*(~*)~*	3052	1713	1339	3084	1527	1557	942	561	381
108	[CH3]~*~*~*~[CH2]~*	3417	2064	1353	2069	1261	808	1101	829	272
109	*~[CH2]~[#8]	3573	2204	1369	2627	1480	1147	964	676	288
110	[#7]~[#6]~[#8]	5235	3226	2009	3891	2185	1706	1452	965	487
111	[#7]~*~[CH2]~*	5287	3400	1887	3938	2343	1595	1422	1060	362
112	*~*(~*)(~*)~*	3757	2245	1512	2998	1597	1401	1267	826	441
113	[#8]!:*:*	5620	3390	2230	5474	3217	2257	1357	852	505
114	[CH3]~[CH2]~*	1871	1214	657	1386	875	511	644	518	126
115	[CH3]~*~[CH2]~*	3523	2183	1340	2352	1279	1073	1447	1005	442
116	[$([CH3]~*~*~[CH2]~*),$([CH3]~*1~*~[CH2]1)]	3739	2338	1401	2319	1372	947	1111	868	243
117	[#7]~*~[#8]	5569	3438	2131	4170	2367	1803	1503	1002	501
118	[$(*~[CH2]~[CH2]~*),$(*1~[CH2]~[CH2]1)]	6000	3635	2365	3979	2314	1665	1664	1219	445
119	[#7]=*	1432	805	627	1121	548	573	291	122	169

120	[!#6;R]	6809	4251	2558	5494	3195	2299	1900	1424	476
121	[#7;R]	7431	4702	2729	6080	3661	2419	1906	1404	502
122	*~[#7](~*)~*	5730	3637	2093	4605	2689	1916	1608	1169	439
123	[#8]~[#6]~[#8]	2958	1650	1308	2472	1455	1017	746	567	179
124	[!#6;!#1]~[!#6;!#1]	3731	2254	1477	3238	1634	1604	910	550	360
125	Special handling required	8451	5188	3263	7414	4257	3157	2266	1493	773
126	*!@[#8]!@*	5620	3313	2307	4778	2790	1988	1446	971	475
127	*@*!@[#8]	7837	4628	3209	6952	4069	2883	1974	1318	656
128	[$(*~[CH2]~*~*~*~[CH2]~*),$([R]1@[CH2;R]@[R]@[R]@[R]@[CH2;R]1)]	5368	3321	2047	3466	1969	1497	1404	1026	378
129	[$(*~[CH2]~*~*~[CH2]~*),$([R]1@[CH2]@[R]@[R]@[CH2;R]1)]	5171	3174	1997	3400	1922	1478	1464	1040	424
130	[!#6;!#1]~[!#6;!#1]	2003	1199	804	2100	1075	1025	527	268	259
131	[!#6;!#1;!H0]	4826	3027	1799	3672	2240	1432	1536	1083	453
132	[#8]~*~[CH2]~*	4735	2897	1838	3332	1824	1508	1554	1060	494
133	*@*!@[#7]	4472	2797	1675	3777	2077	1700	1483	1003	480
134	[F,Cl,Br,I]	3219	1817	1402	3254	1640	1614	974	583	391
135	[#7]!:*:*	4508	2778	1730	3498	1844	1654	1266	817	449
136	[#8]=*	4914	3002	1912	3804	2215	1589	1246	846	400
137	[!C;!c;R]	9016	5522	3494	7181	4262	2919	2334	1703	631
138	[!#6;!#1]~[CH2]~*	4625	3018	1607	3368	2039	1329	1289	955	334
139	[O;!H0]	4049	2457	1592	3275	1953	1322	1297	835	462
140	[#8]	5264	3201	2063	4578	2718	1860	1492	1018	474
141	[CH3]	4144	2641	1503	3172	1933	1239	1252	939	313
142	[#7]	7486	4719	2767	5757	3329	2428	2070	1454	616
143	*@*!@[#8]	7837	4628	3209	6952	4069	2883	1974	1318	656
144	*!:*:*!:*	6856	4196	2660	6030	3542	2488	1738	1140	598
145	*1~*~*~*~*~*~1	9080	5462	3618	7877	4432	3445	2511	1682	829
146	[#8]	7063	4281	2782	6030	3481	2549	1960	1325	635
147	[$(*~[CH2]~[CH2]~*),$([R]1@[CH2;R]@[CH2;R]1)]	6045	3658	2387	3996	2328	1668	1669	1224	445

148	*~[!#6;!#1](~*)~*	6096	3859	2237	5266	3035	2231	1808	1253	555
149	[C;H3,H4]	6237	3949	2288	4777	2839	1938	1770	1323	447
150	*!@*@*!@*	8073	4813	3260	6615	3818	2797	2163	1510	653
151	[#7;!H0]	5990	3753	2237	4546	2642	1904	1656	1134	522
152	[#8]~[#6](~[#6])~[#6]	7928	4745	3183	7284	4275	3009	2327	1606	721
153	[!#6;!#1]~[CH2]~*	6667	4145	2522	5446	3105	2341	1998	1350	648
154	[#6]=[#8]	8233	4896	3337	6432	3572	2860	2409	1683	726
155	*!@[CH2]!@*	6397	3888	2509	5150	2861	2289	1907	1271	636
156	[#7]~*(~*)~*	8582	5347	3235	7384	4307	3077	2349	1583	766
157	[#6]-[#8]	7866	4688	3178	6774	3936	2838	2287	1563	724
158	[#6]-[#7]	7955	4925	3030	6320	3652	2668	2258	1526	732
159	[#8]	9071	5436	3635	7472	4318	3154	2471	1663	808
160	[C;H3,H4]	8304	5112	3192	6742	3795	2947	2556	1777	779
161	[#7]	8912	5545	3367	7624	4439	3185	2496	1685	811
162	Aromatic ring	9765	5891	3874	8440	4813	3627	2741	1828	913
163	*1~*~*~*~*~*~1	10267	6112	4155	8689	4886	3803	2884	1943	941
164	[#8]	10216	6109	4107	8584	4835	3749	2876	1941	935
165	[R]	10542	6295	4247	8924	5020	3904	2953	1994	959

Table S4. Number of fragments obtained from decomposition of the datasets. For each dataset is reported the total number of fragments obtained through the adopted method, and the selected fragments present in at least 20 molecules of the dataset.

Dataset	Number of generated fragments (total)	Number of selected fragments
PC3	157.472	1105
DU-145	138.804	1423
LNCaP	62.595	1043

Table S5. Molecular fragments commonly present in compounds of the PC3, DU-145 or LNCaP datasets, as evaluated with the *custom-fragment* approach.

SMARTS pattern	Molecules matching the SMARTS pattern		
	PC3 dataset	DU-145 dataset	LNCaP
[#6&!H0&!H1&!H2]-[*]	3966	2712	1023
[*]-[#8]-[#6&!H0&!H1&!H2]	3586	3484	880
[*]-[#7&!H0]-[*]	3399	2404	1107
[*]-[#6&!H0&!H1]-[*]	2904	2259	799
[*]-[#8]-[*]	2785	1807	797
[#8&!H0]-[*]	2575	2257	688
[*]-[#6&D3]1:[#6&!H0]:[#6&!H0]:[#6&D3](:[#6&!H0]:[#6&!H0]:1)-[*]	1802	1318	489
[*]-[#6&D3]1:[#6&!H0]:[#6&!H0]:[#6&!H0]:[#6&!H0]:[#6&!H0]:1	1551	1388	374
[#17]-[*]	1329	1340	220
[#9]-[*]	1056	870	245
[*]-[#6&!H0&!H1]-[#6&!H0&!H1]-[*]	974	491	226
[#7&!H0&!H1]-[*]	573	424	117
[*]-[#6&D3]1:[#6&!H0]:[#6&D3](-[*]):[#6&D3](:[#6&D3](:[#6&!H0]:1)-[*])-[*]	525	595	151
[*]-[#6&D3]1:[#6&!H0]:[#6&D3](:[#6&D3](:[#6&D3](:[#6&!H0]:1)-[#8]-[#6&!H0&!H1&!H2])-[*])-[#8]-[#6&!H0&!H1&!H2]	525	594	151
[*]-[#6&D3]1:[#6&!H0]:[#6&D3](:[#6&D3](:[#6&D3](:[#6&!H0]:1)-[#8]-[#6&!H0&!H1&!H2])-[*])-[*]	524	592	151
[*]-[#6&D3]1:[#6&!H0]:[#6&D3](-[*]):[#6&D3](:[#6&D3](:[#6&!H0]:1)-[#8]-[#6&!H0&!H1&!H2])-[#8]-[#6&!H0&!H1&!H2]	520	591	147
[*]-[#6&D3]1:[#6&!H0]:[#6&D3](-[*]):[#6&D3](:[#6&D3](:[#6&!H0]:1)-[*])-[#8]-[#6&!H0&!H1&!H2]	520	590	147
[#35]-[*]	510	537	85
[*]-[#6&D3]1:[#6&!H0]:[#6&!H0]:[#6&D3](:[#6&!H0]:[#6&!H0]:1)-[#8]-[#6&!H0&!H1&!H2]	472	497	72
*-[#6](=[#8])-[#6&!H0&!H1&!H2]	459	339	219
[*]-[#6&!H0&!H1]-[#6&!H0&!H1]-[#6&!H0&!H1]-[*]	428	367	67
[*]-[#7](-[*])-[*]	396	251	56
[*]-[#6](-[#9])(-[#9])-[#9]	371	598	408
[*]-[#6&!H0&!H1]-[#8&!H0]	355	242	101
*-[#7&D3]1-[#6&!H0&!H1]-[#6&!H0&!H1]-[#8&D2]-[#6&!H0&!H1]-[#6&!H0&!H1]-1	354	186	94
[#6&!H0&!H1&!H2]-[#6&!H0&!H1]-[*]	346	268	134
[*]-[#16]-[*]	327	192	110
[*]-[#7](-[#6&!H0&!H1&!H2])-[#6&!H0&!H1&!H2]	310	211	84
[*]-[#7&!H0]-[#6](-[#7&!H0]-[*])=[#8]	309	64	161
[*]-[#8]-[#6](-[#6&!H0&!H1&!H2])=[#8]	260	188	113
[*]-[#6&D3]1:[#6&!H0]:[#6&!H0]:[#6&D3](:[#6&!H0]:[#6&!H0]:1)-[#6&!H0&!H1]-[*]	252	182	82

[*]-[#6](=[#8])-[#8&!H0]	251	174	38
[*]-[#6&!H0&!H1]-[#6&!H0&!H1]-[#7&!H0]-[*]	238	120	34
[#16](=[#8])(=[#8])(-[*])-[*]	224	100	24
[*]-[#8]-[#6](-[*])=[#8]	215	69	36
[*]-[#7](-[*])-[#6&!H0&!H1&!H2]	192	146	21
[*]-[#6&D3]1:[#6&!H0]:[#6&D3](-[*]):[#6&D3](:[#6&!H0]:[#6&!H0]:1)-[#8]-[*]	178	70	31
*-[#7&D3]1-[#6&!H0&!H1]-[#6&!H0&!H1]-[#7&D3](-[#6&!H0&!H1]-[#6&!H0&!H1]-1)-[#6&!H0&!H1&!H2]	163	116	51
[*]-[#6&D3]1:[#6&!H0]:[#6&!H0]:[#6&D3](:[#6&D3](:[#6&!H0]:1)-[#8]-[#6&!H0&!H1&!H2])-[#8]-[*]	144	69	27
[*]-[#6&!H0&!H1]-[#6&!H0&!H1]-[#6&!H0&!H1&!H2]	141	69	33
[#6&!H0&!H1](-*)-[#6&D3]1:[#6&!H0]:[#6&!H0]:[#6&!H0]:[#6&!H0]:[#6&!H0]:1	140	89	31
[#6](#[#7])-[*]	128	254	233
[*]-[#6](-[#6&D3]1:[#6&!H0]:[#6&D3](-[*]):[#6&D3](:[#6&D3](:[#6&!H0]:1)-[*])-[*])=[#8]	120	166	66
[*]-[#6](-[#6&D3]1:[#6&!H0]:[#6&D3](:[#6&D3](:[#6&D3](:[#6&!H0]:1)-[#8]-[#6&!H0&!H1&!H2])-[*])-[#8]-[#6&!H0&!H1&!H2])=[#8]	120	166	66
[*]-[#6](-[#6&D3]1:[#6&!H0]:[#6&D3](:[#6&D3](:[#6&D3](:[#6&!H0]:1)-[#8]-[#6&!H0&!H1&!H2])-[*])-[*])=[#8]	120	165	66
[*]-[#6](-[#6&D3]1:[#6&!H0]:[#6&D3](-[*]):[#6&D3](:[#6&D3](:[#6&!H0]:1)-[#8]-[#6&!H0&!H1&!H2])-[#8]-[#6&!H0&!H1&!H2])=[#8]	120	163	64
[*]-[#6](-[#6&D3]1:[#6&!H0]:[#6&D3](-[*]):[#6&D3](:[#6&D3](:[#6&!H0]:1)-[*])-[#8]-[#6&!H0&!H1&!H2])=[#8]	120	163	64
[*]-[#6&D3]1:[#6&!H0]:[#6&!H0]:[#6&!H0]:[#7&D2]:[#6&!H0]:1	116	77	48
[*]-[#6&!H0&!H1]-[#6&!H0](-[*])-[#6](-[*])=[#8]	112	50	35
[*]-[#6&D3]1:[#6&!H0]:[#6&!H0]:[#6&D3](:[#6&!H0]:[#6&!H0]:1)-[#6](-[#7&!H0]-[*])=[#8]	111	38	22
[#6&!H0](=[#6&!H0]/[*])\[*]	110	80	22
[*]-[#6&D3]1:[#6&!H0]:[#6&!H0]:[#6&D3](:[#6&!H0]:[#6&!H0]:1)-[#7+](=[#8])-[#8-]	98	80	33
[*]-[#6&D3]1:[#6&!H0]:[#6&!H0]:[#7&D2]:[#6&!H0]:[#6&!H0]:1	96	64	27
[*]-[#6&D3]1:[#6&!H0]:[#6&D3](-[*]):[#6&D3](:[#6&!H0]:[#6&!H0]:1)-[#8&!H0]	95	69	81
*-[#7&D3]1-[#6&!H0&!H1]-[#6&!H0&!H1]-[#6&!H0&!H1]-[#6&!H0&!H1]-1	88	63	20
[*]-[#6&!H0&!H1]-[#6](-[#7&!H0]-[*])=[#8]	84	65	23
[*]-[#6](-[#6&D3]1:[#6&!H0]:[#6&D3](-[#8]-[#6&!H0&!H1&!H2]):[#6&D3](:[#6&D3](:[#6&!H0]:1)-[#8]-[#6&!H0&!H1&!H2])-[#8]-[#6&!H0&!H1&!H2])=[#8]	81	94	56

[*]-[#6&!H0&!H1]-[#7&D3]1-[#6&!H0&!H1]-[#6&!H0&!H1]-[#7&D3](-[#6&!H0&!H1]-[#6&!H0&!H1]-1)-[*]	77	50	27
[*]-[#6&D3]1:[#7&D3](-[*]):[#6&!H0]:[#6&!H0]:[#7&D2]:1	75	75	72
[#8](-*)-[#6](-[#6&!H0&!H1&!H2])(-[#6&!H0&!H1&!H2])-[#6&!H0&!H1&!H2]	74	35	22
[*]-[#6&D3]1:[#6&!H0]:[#6&D3](:[#6&D3](:[#6&!H0]:[#6&!H0]:1)-[*])-[#8]-[*]	64	66	26
[*]-[#6&!H0]1-[#6&!H0&!H1]-[#6&!H0&!H1]-[#6&!H0&!H1]-[#6&!H0&!H1]-[#6&!H0&!H1]-1	59	92	23
[*]-[#6&!H0]1-[#7&!H0]-[#6&!H0](-[#6&!H0&!H1]-[#16&D2]-1)-[*]	56	59	69
[*]-[#6&D3]1:[#6&D3](-[#8]-[*]):[#6&D3](:[#6&D3]2:[#6&D3](:[#8&D2]:1):[#6&!H0]:[#6&!H0]:[#6&!H0]:[#6&!H0]:2)=[#8]	56	41	57
[*]-[#6&!H0]1-[#7&!H0]-[#6&!H0](-[#6&!H0&!H1]-[#16&D2]-1)-[#6](-[*])=[#8]	54	57	67
[*]-[#6&!H0]1-[#7&!H0]-[#6&!H0](-[#6&!H0&!H1]-[#16&D2]-1)-[#6](-[#7&!H0]-[*])=[#8]	51	54	63
[*]-[#6&D3]1:[#6&!H0]:[#6&D3](-[*]):[#6&!H0]:[#6&D3](:[#6&!H0]:1)-[#6](-[#9])(-[#9])-[#9]	51	43	41
[*]-[#6&D3]1:[#6&D3](-[#8]-[*]):[#6&!H0]:[#6&!H0]:[#6&!H0]:[#6&!H0]:1	51	37	20
[*]-[#6&!H0&!H1]-[#6&!H0](-[#7&!H0]-[*])-[#6](-[*])=[#8]	50	23	27
[*]-[#6&D3]1:[#6&!H0]:[#6&D3](-[#6](-[#9])(-[#9])-[#9]):[#6&!H0]:[#6&D3](:[#6&!H0]:1)-[#6](-[#9])(-[#9])-[#9]	49	40	41
[*]-[#6&D3]1:[#6&!H0]:[#6&D3](-[*]):[#6&!H0]:[#6&D3](:[#6&!H0]:1)-[#8]-[#6&!H0&!H1&!H2]	44	88	20
[*]-[#6&!H0](-[#6](-[*])=[#8])-[#6&!H0](-[#6&!H0&!H1&!H2])-[#6&!H0&!H1&!H2]	39	213	119
[*]-[#6&!H0](-[*])-[*]	39	44	27
[*]-[#6&D3]1:[#6&!H0]:[#6&D3](-[#6&D3]2:[#6&D3](-[*]):[#6&D3](:[#6&D3]3:[#6&D3](:[#8&D2]:2):[#6&!H0]:[#6&!H0]:[#6&!H0]:[#6&!H0]:3)=[#8]):[#6&!H0]:[#6&!H0]:[#6&D3]:1-[#8]-[#6&!H0&!H1&!H2]	37	31	38
[*]-[#6&D3]1:[#6&!H0]:[#6&!H0]:[#6&D3](:[#6&!H0]:[#6&D3]:1-[#8]-[#6&!H0&!H1&!H2])-[#6&D3]1:[#6&D3](-[#8]-[*]):[#6&D3](:[#6&D3]2:[#6&D3](:[#8&D2]:1):[#6&!H0]:[#6&!H0]:[#6&!H0]:[#6&!H0]:2)=[#8]	36	30	37
[*]-[#6&D3]1:[#6&!H0]:[#6&D3](-[#6&D3]2:[#6&D3](-[#8]-[*]):[#6&D3](:[#6&D3]3:[#6&D3](:[#8&D2]:2):[#6&!H0]:[#6&!H0]:[#6&!H0]:[#6&!H0]:3)=[#8]):[#6&!H0]:[#6&!H0]:[#6&D3]:1-[#8]-[#6&!H0&!H1&!H2]	36	30	37
[*]-[#6&D3]1:[#6&D3](-[*]):[#6&!H0]:[#6&D3](:[#6&!H0]:[#6&!H0]:1)-[#6&D3]1:[#6&D3](-[#8]-	36	30	37

[*]):[#6&D3](:[#6&D3]2:[#6&D3](:[#8&D2]:1):[#6&!H0]:[#6&!H0]:[#6&!H0]:[#6&!H0]:2)=[#8]			
[*]-[#6&!H0]1-[#7&D3](-[*])-[#6&!H0&!H1]-[#6&!H0&!H1]-[#6&!H0&!H1]-1	35	144	97
[*]-[#6&D3]1:[#6&!H0]:[#6&!H0]:[#6&D3](:[#6&!H0]:[#6&!H0]:1)-[#6&!H0&!H1]-[#7&D3]1-[#6&!H0&!H1]-[#6&!H0&!H1]-[#7&D3](-[#6&!H0&!H1]-[#6&!H0&!H1]-1)-[*]	35	30	21
*-[#7&!H0]-[#6&!H0&!H1]-[#6&!H0&!H1]-[#6&!H0&!H1]-[#6&!H0&!H1]-[#6&!H0&!H1]-[#6&!H0&!H1]-[#6&!H0&!H1]-[#6&!H0&!H1]-[#6&!H0&!H1]-[#6&!H0&!H1]-[#6&!H0&!H1]-[#6&!H0&!H1]-[#6&!H0&!H1]-[#6&!H0&!H1]-[#6&!H0&!H1]-[#6&!H0&!H1]-[#6&!H0&!H1]-[#6&!H0&!H1&!H2]	34	38	42
[#6&D3]1(-*):[#7&D2]:[#6&D3](-*):[#7&D2]:[#6&D3](:[#7&D2]:1)-*	34	26	45
[*]-[#8]-[#6](-[#9])(-[#9])-[#9]	33	60	57
[*]-[#6&D3]1:[#6&!H0]:[#6&!H0]:[#6&D3](:[#6&!H0]:[#6&!H0]:1)-[#6&!H0&!H1]-[#8]-[*]	32	56	39
[*]-[#6&!H0](-[#6&!H0&!H1]-[#6&!H0&!H1&!H2])-[#6&!H0&!H1&!H2]	30	29	25
[*]-[#6&!H0](-[#6](-[#7&!H0]-[*])=[#8])-[#6&!H0](-[#6&!H0&!H1&!H2])-[#6&!H0&!H1&!H2]	29	137	68
[*]-[#6&D3]1:[#7&D2]:[#6&D3](:[#6&!H0]:[#16&D2]:1)-[#6](-[*])=[#8]	28	24	25
[#6&!H0]1(-[#6&!H0&!H1]-[#6&!H0](-[#6&!H0](-[#6&!H0](-[#8&D2]-1)-[*])-[*])-[*])-[*]	27	27	27
[*]-[#6&D3]1:[#6&!H0]:[#6&D3]2-[#8&D2]-[#6&!H0](-[*])-[#6&!H0](-[#8&D2]-[#6&D3]:2:[#6&!H0]:[#6&!H0]:1)-[*]	26	25	57
[*]-[#6&!H0](-[*])-[#6](-[*])=[#8]	25	64	20
[*]-[#6&D3]1:[#6&!H0]:[#6&!H0]:[#6&D3](:[#6&!H0]:[#6&D3]:1-[#8]-[#6&!H0&!H1&!H2])-[#6&D3]1:[#6&D3](-[*]):[#6&D3](:[#6&D3]2:[#6&D3](:[#8&D2]:1):[#6&!H0]:[#6&D3](:[#6&!H0]:[#6&!H0]:2)-[#8]-[#6&!H0&!H1&!H2])=[#8]	25	21	25
[*]-[#6&D3]1:[#6&!H0]:[#6&D3](-[#6&D3]2:[#6&D3](-[*]):[#6&D3](:[#6&D3]3:[#6&D3](:[#8&D2]:2):[#6&!H0]:[#6&D3](:[#6&!H0]:[#6&!H0]:3)-[#8]-[#6&!H0&!H1&!H2])=[#8]):[#6&!H0]:[#6&!H0]:[#6&D3]:1-[#8]-[#6&!H0&!H1&!H2]	25	21	25
[*]-[#6&D3]1:[#6&D3](-[*]):[#6&!H0]:[#6&D3](:[#6&!H0]:[#6&!H0]:1)-[#6&D3]1:[#6&D3](-[*]):[#6&D3](:[#6&D3]2:[#6&D3](:[#8&D2]:1):[#6&!H0]:[#6&D3](:[#6&!H0]:[#6&!H0]:2)-[#8]-[#6&!H0&!H1&!H2])=[#8]	25	21	25
[*]-[#6&D3]1:[#6&!H0]:[#6&!H0]:[#6&D3](:[#6&!H0]:[#6&D3]:1-[#8]-[#6&!H0&!H1&!H2])-[#6&D3]1:[#6&D3](-[#8]-[*]):[#6&D3](:[#6&D3]2:[#6&D3](:[#8&D2]:1):[#6&!H0]:[#6&D3](:[#6&!H0]:[#6&!H0]:2)-[#8]-[#6&!H0&!H1&!H2])=[#8]	24	20	24

[*]-[#6&D3]1:[#6&!H0]:[#6&D3](-[#6&D3]2:[#6&D3](-[#8]-[*]):[#6&D3](:[#6&D3]3:[#6&D3](:[#8&D2]:2):[#6&!H0]:[#6&D3](:[#6&!H0]:[#6&!H0]:3)-[#8]-[#6&!H0&!H1&!H2])=[#8]):[#6&!H0]:[#6&!H0]:[#6&D3]:1-[#8]-[#6&!H0&!H1&!H2]	24	20	24
[*]-[#6&D3]1:[#6&!H0]:[#6&D3]2:[#8&D2]:[#6&D3](-[#6&D3]3:[#6&!H0]:[#6&D3](-[*]):[#6&D3](:[#6&!H0]:[#6&!H0]:3)-[#8]-[#6&!H0&!H1&!H2]):[#6&D3](:[#6&D3](:[#6&D3]:2:[#6&!H0]:[#6&!H0]:1)=[#8])-[#8]-[*]	24	20	24
[*]-[#6&D3]1:[#6&!H0]:[#6&D3]2:[#8&D2]:[#6&D3](-[#6&D3]3:[#6&!H0]:[#6&D3](:[#6&D3](:[#6&!H0]:[#6&!H0]:3)-[*])-[*]):[#6&D3](:[#6&D3](:[#6&D3]:2:[#6&!H0]:[#6&!H0]:1)=[#8])-[#8]-[*]	24	20	24
[*]-[#6&D3]1:[#6&D3](-[#8]-[*]):[#6&D3](:[#6&D3]2:[#6&D3](:[#8&D2]:1):[#6&!H0]:[#6&D3](:[#6&!H0]:[#6&!H0]:2)-[#8]-[#6&!H0&!H1&!H2])=[#8]	24	20	24
[*]-[#6&D3]1:[#6&D3](-[*]):[#6&!H0]:[#6&D3](:[#6&!H0]:[#6&!H0]:1)-[#6&D3]1:[#6&D3](-[#8]-[*]):[#6&D3](:[#6&D3]2:[#6&D3](:[#8&D2]:1):[#6&!H0]:[#6&D3](:[#6&!H0]:[#6&!H0]:2)-[#8]-[#6&!H0&!H1&!H2])=[#8]	24	20	24
[*]-[#6&D3]1:[#8&D2]:[#6&D3]2:[#6&!H0]:[#6&D3](-[*]):[#6&!H0]:[#6&!H0]:[#6&D3]:2:[#6&D3](:[#6&D3]:1-[#8]-[*])=[#8]	24	20	24
[*]-[#6](-[#7&!H0]-[#6&!H0&!H1]-[#6&!H0&!H1]-[#6&!H0&!H1]-[#6&!H0&!H1]-[#6&!H0&!H1]-[#6&!H0&!H1]-[#6&!H0&!H1]-[#6&!H0&!H1]-[#6&!H0&!H1]-[#6&!H0&!H1]-[#6&!H0&!H1]-[#6&!H0&!H1]-[#6&!H0&!H1]-[#6&!H0&!H1]-[#6&!H0&!H1]-[#6&!H0&!H1]-[#6&!H0&!H1]-[#6&!H0&!H1&!H2])=[#8]	23	25	31
[*]-[#6&!H0]1-[#16&D2]-[#6&!H0&!H1]-[#6&!H0](-[#7&!H0]-1)-[#6](-[#7&!H0]-[#6&!H0&!H1]-[#6&!H0&!H1]-[#6&!H0&!H1]-[#6&!H0&!H1]-[#6&!H0&!H1]-[#6&!H0&!H1]-[#6&!H0&!H1]-[#6&!H0&!H1]-[#6&!H0&!H1]-[#6&!H0&!H1]-[#6&!H0&!H1]-[#6&!H0&!H1]-[#6&!H0&!H1]-[#6&!H0&!H1]-[#6&!H0&!H1]-[#6&!H0&!H1]-[#6&!H0&!H1]-[#6&!H0&!H1&!H2])=[#8]	23	25	30
[*]-[#6&D3]1:[#7&D2]:[#6&D3](:[#6&!H0]:[#16&D2]:1)-[#6](-[#6&D3]1:[#6&!H0]:[#6&D3](:[#6&D3](:[#6&D3](:[#6&!H0]:1)-[#8]-[#6&!H0&!H1&!H2])-[*])-[#8]-[#6&!H0&!H1&!H2])=[#8]	22	27	20
[*]-[#6&D3]1:[#7&D2]:[#6&D3](:[#6&!H0]:[#16&D2]:1)-[#6](-[#6&D3]1:[#6&!H0]:[#6&D3](:[#6&D3](:[#6&D3](:[#6&!H0]:1)-[#8]-[#6&!H0&!H1&!H2])-[*])-[*])=[#8]	22	26	20
*-[#7&D3]1-[#6&!H0&!H1]-[#6&!H0&!H1]-[#7&D3](-[#6&!H0&!H1]-[#6&!H0&!H1]-1)-[#6&D3]1:[#6&!H0]:[#6&!H0]:[#6&!H0]:[#6&!H0]:[#6&!H0]:1	21	36	22

Table S6. Hyperparameters configurations and number of features retained for each ML model trained in the PC3, DU-145 and LNCaP datasets.

		PC3				***DU-145***				***LNCAP***			
ET		**MACCS keys**	**RDKit's descriptors**	**ECFP4 fin-gerprints**	***Custom-fragments***	**MACCS keys**	**RDKit's descriptors**	**ECFP4 fin-gerprints**	***Custom-fragments***	**MACCS keys**	**RDKit's descriptors**	**ECFP4 fin-gerprints**	***Custom-fragments***
N° of features retained		28	108	735	500	15	196	41	1301	73	50	876	878
N° estimators	100; 150; 200; 250; 300; 350; 400; 450; 500	250	500	400	400	300	400	300	300	100	150	500	200
Max depth	5; 10; 15	15	15	15	15	15	15	15	15	10	15	15	15
Min samples split	10; 20; 30	10	10	10	10	10	10	10	10	10	10	30	10
Min samples leaf	10; 15; 20	10	10	10	10	10	10	10	10	10	10	10	10
Max features	sqrt; log2; None (N)	sqrt	N	sqrt	N	sqrt	N	sqrt	N	sqrt	N	N	N
Bootstrap	True (T); False (F)	F	F	F	T	F	F	F	T	F	F	T	F
Criterion	Gini (G); entropy (E)	E	E	G	G	E	G	G	G	G	G	E	G
Class weight	None (N); balanced (B)	B	B	B	B	N	B	B	B	B	N	B	B
RF		**MACCS keys**	**RDKit's descriptors**	**ECFP4 fin-gerprints**	***Custom-fragments***	**MACCS keys**	**RDKit's descriptors**	**ECFP4 fin-gerprints**	***Custom-fragments***	**MACCS keys**	**RDKit's descriptors**	**ECFP4 fin-gerprints**	***Custom-fragments***
N° of features retained		35	90	838	975	16	180	627	801	23	114	11	531
N° estimators	100; 150; 200; 250; 300; 350; 400; 450; 500	150	200	400	400	200	400	400	300	500	250	250	100
Max depth	5; 10; 15	15	15	15	15	15	15	15	15	15	15	15	15
Min samples split	10; 20; 30	10	10	10	10	10	30	30	10	30	10	10	30
Min samples leaf	10; 15; 20	10	10	10	10	10	10	10	10	10	10	10	10
Max features	sqrt; log2; None (N)	N	sqrt	sqrt	N	sqrt	sqrt	sqrt	N	sqrt	log2	N	sqrt
Bootstrap	True (T); False (F)	T	F	F	T	F	F	F	T	F	F	T	T
Criterion	Gini (G); entropy (E)	G	E	E	E	G	G	E	G	E	G	E	G
Class weight	None (N); balanced (B)	B	B	B	B	B	B	B	B	B	B	B	B
GBM		**MACCS keys**	**RDKit's descriptors**	**ECFP4 fin-gerprints**	***Custom-fragments***	**MACCS keys**	**RDKit's descriptors**	**ECFP4 fin-gerprints**	***Custom-fragments***	**MACCS keys**	**RDKit's descriptors**	**ECFP4 fin-gerprints**	***Custom-fragments***
N° of features retained		123	130	795	503	14	182	774	501	16	83	931	874
N° estimators	250; 300; 350	350	350	300	350	300	350	350	350	300	300	350	350
Learning rate	0.03; 0.04; 0.05; 0.07	0.05	0.04	0.05	0.05	0.04	0.04	0.05	0.07	0.05	0.04	0.05	0.05
Max depth	6 ;7; 8; 9	7	7	7	7	7	7	7	9	7	7	7	7
Min samples split	10; 15; 20	15	15	15	15	15	15	15	15	15	15	15	15
Min samples leaf	10; 15; 20	15	15	15	15	15	20	15	15	15	15	20	20
Subsample	0.7; 0.8;0.9	0.8	0.9	0.9	0.8	0.9	0.9	0.9	0.9	0.9	0.9	0.8	0.9

Max features	Sqrt; None (N)	sqrt	sqrt	sqrt	sqrt	sqrt	sqrt	sqrt	N	sqrt	sqrt	sqrt	sqrt
XGB		**MACCS keys**	**RDKit's descriptors**	**ECFP4 fingerprints**	***Custom-fragments***	**MACCS keys**	**RDKit's descriptors**	**ECFP4 fingerprints**	***Custom-fragments***	**MACCS keys**	**RDKit's descriptors**	**ECFP4 fingerprints**	***Custom-fragments***
N° of features retained		34	90	590	852	15	134	742	1380	12	82	786	797
N° estimators	150; 200; 250; 300	250	150	250	300	150	150	250	250	250	150	250	150
Max depth	5; 8; 9; 10	8	5	8	9	8	5	8	10	8	5	8	8
Learning rate	0.05; 0.06; 0.07; 0.1	0.07	0.05	0.07	0.1	0.05	0.05	0.07	0.06	0.07	0.05	0.07	0.07
Subsample	0.7; 0.8; 0.9	0.9	0.6	0.7	0.7	0.7	0.6	0.7	0.9	0.9	0.8	0.7	0.9
Colsample by tree	0.7; 0.8; 0.9	0.8	0.8	0.8	0.7	0.7	0.6	0.8	0.7	0.8	0.8	0.7	0.8
Gamma	0; 0.1; 0.3; 0.5	0	0.1	0.1	0.3	0	0	0	0.1	0.1	0.3	0.5	0.1
Min child weight	1; 3; 5	1	1	1	1	1	1	1	1	1	5	1	1
Reg lambda	0.5; 1; 1.5	1.25	1	1.25	1.25	1.25	1	1.25	1	1.25	1	1.25	1.25
Reg alpha	0; 0.25; 0.5	0.75	0	0.5	0	0.5	0.5	0.5	0.25	0.5	0.5	0.75	0.5
Scale pos weight	1; 1.5; 2	1	1	1	1	1	1	1	1	1	1	1	1

Table S7. RDKit's descriptors commonly retained by ML models trained on PC3, DU-145 and LNCaP datasets after RFEcv. RDKit's descriptors retained in the trained models are marked by "X".

RDKit's descriptors	Category	Subcategory	PC3				DU-145				LNCaP			
			ET	RF	GBM	XGB	ET	RF	GBM	XGB	ET	RF	GBM	XGB
MaxAbsEStateIndex	Electronic / Charge Descriptors	EState Descriptors	X	X	X	X	X	X	X	X		X	X	X
MaxEStateIndex			X	X	X		X	X	X	X		X	X	
MinAbsEStateIndex			X	X	X		X	X	X	X		X	X	
MinEStateIndex			X	X	X	X	X	X	X	X	X	X	X	X
MaxPartialCharge		Partial Charge Descriptors	X	X	X	X	X	X	X	X		X	X	X
MinPartialCharge			X	X	X	X	X	X	X	X		X	X	X
MaxAbsPartialCharge			X	X	X	X	X	X	X	X		X	X	X
MinAbsPartialCharge			X	X	X	X	X	X	X	X		X	X	X
EState_VSA1		EState VSA Descriptors		X	X	X	X	X	X	X	X	X	X	
EState_VSA2			X	X	X	X	X	X	X	X		X		
EState_VSA3					X	X	X	X	X	X				
EState_VSA4							X	X	X	X				
EState_VSA5							X		X					
EState_VSA6							X	X	X					
EState_VSA7								X						
EState_VSA8			X	X	X	X	X	X	X	X		X	X	X
EState_VSA9			X	X	X	X	X	X	X	X		X	X	
EState_VSA10				X	X		X	X	X	X		X		X
EState_VSA11							X	X	X					
VSA_EState1		VSA EState Descriptors		X	X		X	X	X	X		X		X
VSA_EState2			X	X	X	X	X	X	X	X		X	X	X
VSA_EState3			X	X	X	X	X	X	X	X		X	X	
VSA_EState4			X	X	X	X	X	X	X	X		X	X	X
VSA_EState5				X	X	X	X	X	X	X		X	X	
VSA_EState6			X	X	X	X	X	X	X	X	X	X	X	X
VSA_EState7				X	X	X	X	X	X	X	X	X	X	X
VSA_EState8			X	X	X	X	X	X	X	X		X	X	
VSA_EState9			X	X	X	X	X	X	X	X		X	X	X
VSA_EState10					X		X	X	X	X				X
MolWt	Constitutional Descriptors	Basic Molecular Properties		X	X		X	X	X	X		X	X	
HeavyAtomMolWt					X		X	X	X	X		X	X	X
ExactMolWt					X		X	X	X			X	X	
NumValenceElectrons					X		X	X	X	X		X		
NumRadicalElectrons							X	X	X					

HeavyAtomCount		Atom and Ring Counts	X		X		X	X	X		X	X		
FractionCSP3			X	X	X	X	X	X	X	X	X	X	X	X
NHOHCount			X		X		X	X	X	X	X	X		
NOCount			X	X	X		X	X	X	X				
NumHAcceptors			X	X	X	X	X	X	X	X		X		X
NumHDonors			X		X		X	X	X	X		X		X
NumHeteroatoms			X		X		X	X	X			X		
NumRotatableBonds			X		X	X	X	X	X	X		X		
NumAliphaticCarbocycles			X				X	X	X					
NumAliphaticHeterocycles			X	X	X	X	X	X	X		X	X		
NumAliphaticRings			X		X	X	X	X	X	X		X		
NumAromaticCarbocycles			X		X	X	X	X	X					
NumAromaticHeterocycles			X		X	X	X	X	X	X	X	X	X	X
NumAromaticRings			X				X	X	X		X	X		X
NumSaturatedCarbocycles						X	X	X	X					
NumSaturatedHeterocycles			X		X		X	X	X		X			X
NumSaturatedRings			X		X	X	X	X	X	X				X
RingCount			X		X	X	X	X	X	X		X		
Chi0	Topological Descriptors	Chi (Kier–Hall) Indices		X	X		X	X	X	X		X	X	
Chi0n				X	X	X	X	X	X	X	X	X	X	X
Chi0v					X		X	X	X	X		X		
Chi1				X	X	X	X	X	X	X	X	X	X	
Chi1n				X	X		X	X	X	X	X	X	X	X
Chi1v				X	X		X	X	X			X	X	X
Chi2n			X	X	X		X	X	X	X	X	X	X	
Chi2v				X	X		X	X	X	X		X	X	
Chi3n				X	X		X	X	X	X	X	X	X	
Chi3v			X	X	X		X	X	X	X		X	X	X
Chi4n			X	X	X	X	X	X	X	X	X	X	X	X
Chi4v			X	X	X	X	X	X	X	X		X	X	X
Kappa1		Kappa (shape) Indices		X	X		X	X	X	X		X	X	X
Kappa2			X	X	X	X	X	X	X	X		X	X	
Kappa3			X	X	X	X	X	X	X	X		X	X	
HallKierAlpha		Hall–Kier Alpha	X	X	X	X	X	X	X	X		X	X	X
BalabanJ		Balaban's J	X	X	X	X	X	X	X	X		X	X	X
BertzCT		Bertz Complexity		X	X	X	X	X	X	X	X	X	X	X
AvgIpc		Information Content	X	X	X	X	X	X	X	X	X	X	X	X
BCUT2D_MWHI	BCUT (2D) Descriptors	BCUT2D												
BCUT2D_MWLOW														
BCUT2D_CHGHI														

BCUT2D_CHGLO														
BCUT2D_LOGPHI														
BCUT2D_LOGPLOW														
BCUT2D_MRHI														
BCUT2D_MRLOW														
3d_Asphericity			X	X	X		X	X	X		X	X	X	X
3d_Eccentricity				X	X		X	X	X	X		X	X	X
3d_InertialShapeFactor			X	X	X	X	X	X	X	X	X	X	X	X
3d_NPR1			X	X	X		X	X	X	X		X	X	
3d_NPR2			X	X	X		X	X	X	X		X	X	
3d_PBF	3D Geometric / Shape Descriptors	3D Shape / Geometry	X	X	X		X	X	X	X		X	X	
3d_PMI1				X	X	X	X	X	X	X		X	X	X
3d_PMI2				X	X		X	X	X	X		X	X	X
3d_PMI3					X		X	X	X	X		X	X	
3d_RadiusOfGyration				X	X		X	X	X	X		X	X	X
3d_SpherocityIndex			X	X	X		X	X	X	X		X	X	X
LabuteASA		Labute ASA		X	X	X	X	X	X	X	X	X	X	
PEOE_VSA1			X	X	X	X	X	X	X	X		X		
PEOE_VSA2			X	X	X	X	X	X	X	X		X		X
PEOE_VSA3			X	X	X	X	X	X	X	X	X	X	X	X
PEOE_VSA4					X		X	X	X	X				X
PEOE_VSA5					X	X	X	X	X	X				
PEOE_VSA6			X	X	X	X	X	X	X	X		X	X	
PEOE_VSA7			X	X	X		X	X	X	X		X	X	X
PEOE_VSA8		PEOE_VSA	X	X	X	X	X	X	X	X		X	X	
PEOE_VSA9			X	X	X	X	X	X	X	X		X	X	X
PEOE_VSA10			X	X	X	X	X	X	X	X		X		
PEOE_VSA11			X	X	X	X	X	X	X	X	X	X	X	X
PEOE_VSA12	Surface Area & VSA (MOE-type) Descriptors		X	X	X	X	X	X	X	X		X	X	
PEOE_VSA13			X		X	X	X	X	X	X				X
PEOE_VSA14			X		X		X	X	X	X		X		X
SMR_VSA1				X	X		X	X	X	X		X	X	X
SMR_VSA2							X	X	X	X				
SMR_VSA3			X	X	X	X	X	X	X	X	X	X	X	X
SMR_VSA4			X	X	X	X	X	X	X	X		X	X	X
SMR_VSA5		SMR_VSA	X	X	X		X	X	X		X	X	X	X
SMR_VSA6			X	X	X	X	X	X	X	X	X	X	X	X
SMR_VSA7			X	X	X	X	X	X	X	X	X	X	X	X
SMR_VSA8														
SMR_VSA9			X	X	X	X	X	X	X	X		X		

SMR_VSA10			X	X	X	X	X	X	X	X		X	X	X
SlogP_VSA1		SlogP_VSA	X	X	X		X	X	X	X		X	X	X
SlogP_VSA2			X	X	X	X	X	X	X	X		X	X	X
SlogP_VSA3			X	X	X	X	X	X	X	X		X		
SlogP_VSA4			X		X	X	X	X	X	X	X	X		X
SlogP_VSA5			X	X	X	X	X	X	X	X	X	X	X	X
SlogP_VSA6			X	X	X	X	X	X	X	X		X	X	X
SlogP_VSA7					X		X	X	X	X	X	X		X
SlogP_VSA8			X	X	X	X	X	X	X	X	X	X	X	X
SlogP_VSA9														
SlogP_VSA10			X	X	X	X	X	X	X	X	X	X	X	X
SlogP_VSA11			X		X	X	X	X	X	X				
SlogP_VSA12					X		X	X	X	X				
FpDensityMorgan1	Fingerprint Density Descriptors	Morgan Fingerprint Density	X	X	X		X	X	X	X	X	X	X	X
FpDensityMorgan2			X	X	X	X	X	X	X	X	X	X	X	X
FpDensityMorgan3			X	X	X	X	X	X	X	X		X	X	X
qed	Drug-likeness & Physicochemical Descriptors	General Drug-likeness Descriptors	X	X	X		X	X	X	X		X	X	X
SPS			X	X	X	X	X	X	X	X	X	X	X	X
TPSA			X	X	X	X	X	X	X	X		X	X	
MolLogP			X	X	X		X	X	X	X		X	X	X
MolMR			X	X	X	X	X	X	X	X	X	X	X	X
fr_Al_COO	Constitutional Descriptors	Functional Group / Substructure					X		X				X	
fr_Al_OH							X	X	X	X				
fr_Al_OH_noTert						X	X							
fr_ArN							X	X	X	X				
fr_Ar_COO							X		X					
fr_Ar_N			X		X		X	X	X	X	X	X		X
fr_Ar_NH						X	X	X	X	X				
fr_Ar_OH							X	X	X					
fr_COO						X	X		X	X				X
fr_COO2							X		X					
fr_C_O			X		X		X	X	X	X				
fr_C_O_noCOO			X				X	X	X		X			
fr_C_S							X	X	X	X				
fr_HOCCN								X						
fr_Imine							X	X	X					
fr_NH0			X		X	X	X	X	X	X	X	X		X
fr_NH1			X			X	X	X	X	X	X			
fr_NH2							X	X	X	X	X			X
fr_N_O			X		X	X	X	X	X					

fr_Ndealkylation1							X		X					
fr_Ndealkylation2							X	X	X					
fr_Nhpyrrole							X	X	X					
fr_SH							X	X						
fr_aldehyde						X			X					
fr_alkyl_carbamate							X							
fr_alkyl_halide							X	X	X		X	X		X
fr_allylic_oxid			X		X	X	X	X	X	X	X	X		X
fr_amide			X		X	X	X	X	X	X				
fr_amidine							X		X					
fr_aniline			X		X	X	X	X	X	X				
fr_aryl_methyl			X				X	X	X		X			X
fr_azide							X	X	X					
fr_azo							X	X						
fr_barbitur														
fr_benzene			X		X		X	X	X		X			
fr_benzodiazepine														
fr_bicyclic			X		X		X	X	X	X				
fr_diazo														
fr_dihydropyridine							X	X						
fr_epoxide							X	X	X	X				
fr_ester							X	X	X	X				
fr_ether			X		X		X	X	X	X				
fr_furan			X		X	X	X	X						
fr_guanido							X		X			X		
fr_halogen							X	X	X	X				
fr_hdrzine							X		X					
fr_hdrzone							X		X					
fr_imidazole			X				X	X	X	X	X	X		X
fr_imide						X	X	X	X	X				
fr_isocyan														
fr_isothiocyan							X	X						
fr_ketone			X		X	X	X	X	X	X	X	X		X
fr_ketone_Topliss							X	X	X	X				
fr_lactam							X	X						
fr_lactone			X			X	X		X					
fr_methoxy			X				X	X	X					
fr_morpholine							X		X		X	X		X
fr_nitrile							X	X	X					
fr_nitro							X							

fr_nitro_arom							X							
fr_nitro_arom_nonortho														
fr_nitroso							X	X						
fr_oxazole								X						
fr_oxime							X	X	X	X				
fr_para_hydroxylation			X				X	X	X					
fr_phenol							X	X	X					
fr_phenol_noOrthoHbond							X	X	X					
fr_phos_acid								X	X					
fr_phos_ester								X						
fr_piperdine							X		X					
fr_piperzine							X	X	X	X				
fr_priamide							X							
fr_prisulfonamd														
fr_pyridine			X	X	X	X	X	X	X	X				
fr_quatN			X				X	X						
fr_sulfide			X		X	X	X		X					
fr_sulfonamd							X		X					
fr_sulfone							X	X	X					
fr_term_acetylene								X						
fr_tetrazole							X	X						
fr_thiazole						X	X		X					
fr_thiocyan							X	X						
fr_thiophene							X	X	X	X				
fr_unbrch_alkane														
fr_urea			X		X	X	X				X	X		X

Table S8. Statistical comparisons of significative differences between aggregated training cross-validation-fold performance metrics. Friedman's tests were first conducted on all datasets values, then specific comparisons were performed between pairs; Wilcoxon pairwise *p*-values were adjusted according to the Bonferroni's corrections; the effect's size was also evaluated.

MCC Statistical tests	Friedman's test	Comparisons	Wilcoxon's pairwise test	Effect's size
		**Feature-level aggregation**		
**DU-145**	*p value = 3.49x10-6*			
		ecfp4 vs custom_fragments	*0.009*	*0.84*
		ecfp4 vs maccs	*0.012*	*0.89*
		Custom_fragments vs maccs	*0.010*	*0.86*
		custom_fragments vs RDKit	*0.010*	*0.86*
		maccs vs RDKit	*0.011*	*0.88*
**LNCaP**	*p value = 7.04x10-5*			
		ecfp4 vs maccs	*0.014*	*0.87*
		custom_fragments vs RDKit	*0.023*	*0.84*
		maccs vs RDKit	*0.011*	*0.88*
**PC3**	*p value = 7.45x10-5*			
		ecfp4 vs custom fragment	*0.011*	*0.89*
		custom_fragments vs maccs	*0.014*	*0.87*
		custom_fragments vs RDKit	*0.012*	*0.88*
		**Algorithm-level aggregation**		
**DU-145**	*p value = 0.0003*			
		ET vs RF	*0.011*	*0.88*
		XGB vs ET	*0.012*	*0.89*
**LNCaP**	*p value = 0.006*			
		ET vs RF	*0.033*	*0.88*
		XGB vs ET	*0.010*	*0.74*
**PC3**	*p value = 0.0001*			
		XGB vs ET	*0.011*	*0.89*
		XGB vs GBM	*0.014*	*0.87*
		XGB vs RF	*0.012*	*0.88*

Table S9. Selected top RDKit's molecular descriptors by "RAW" and SHAP values most frequently emerging from misclassified compounds of each PC test set. The table reports the percentages of misclassified and correctly compounds with feature values in the opposite class ranges, the averaged absolute SHAP contributions, overall SHAP feature ranking, and count of clusters where each feature has no overlapping "RAW" and SHAP values.

PC3 features	Averaged absolute SHAP contribution	Ranking of features based on SHAP values	% of misclassified compounds with opposite class ranges of values		% of correctly classified compounds with opposite-class ranges of values		Number of clusters with non-overlapping class values	
			"RAW"	SHAP	"RAW"	SHAP	"RAW"	SHAP
VSA_ESTATE4	0.04979	21	12.40	10.82	5.26	4.23	212	202
fr_furan	0.00844	125	31.93	12.14	19.94	4.39	8	208
BALABANJ	0.06462	9	11.74	12.01	5.39	4.62	207	207
fr_allylic_oxid	0.01737	92	30.21	12.14	17.15	4.71	38	190
AVGIPC	0.06240	10	11.61	10.69	5.71	4.78	209	207
SPS	0.02882	55	11.35	11.87	6.57	4.78	202	201
Estate_VSA9	0.04722	25	12.53	10.29	6.12	4.81	190	207
MaxAbsPartialCharge	0.04613	27	15.96	11.74	7.21	4.81	168	214
fr_pyridine	0.08086	5	31.13	12.93	18.63	4.81	21	196
SMR_VSA1	0.03619	40	13.19	12.80	5.23	4.84	195	200
BERTZCT	0.01964	84	11.61	12.40	5.29	4.87	203	195
fr_c_o	0.00761	127	25.07	13.06	14.27	4.87	74	204
fr_amide	0.01324	109	27.70	11.61	16.26	4.87	45	209
TPSA	0.02989	51	15.96	12.27	6.76	4.94	174	199
SMR_VSA10	0.05479	13	17.81	11.21	8.30	4.97	142	202
VSA_Estate6	0.14172	1	12.14	10.55	4.84	5.00	209	208
CHI4v	0.04384	29	12.40	10.69	5.16	5.00	215	224
VSA_Estate2	0.03664	38	12.01	11.35	5.16	5.03	216	215
SlogP_VSA8	0.12729	2	23.88	9.89	14.20	5.03	79	216
SMR_VSA6	0.04851	24	20.05	11.48	10.10	5.07	123	217

DU-145 features	Averaged absolute SHAP contribution	Ranking of features based on SHAP values	% of misclassified compounds with opposite-class ranges of values		% of correctly classified compounds with opposite-class ranges of values		Number of clusters with non-overlapping class values	
			"RAW"	SHAP	"RAW"	SHAP	"RAW"	SHAP
SMR_VSA10	0.03849	28	25.59	21.06	15.01	9.82	69	74
fr_bicyclic	0.05228	8	32.29	19.03	22.92	10.05	33	83
numaromaticheterocycles	0.00633	144	31.51	19.66	23.38	10.05	21	82
Estate_VSA4	0.00869	136	34.63	20.59	28.07	10.12	7	79
fr_phenol_noorthohbond	0.01255	119	33.39	19.34	26.43	10.16	11	83
PEOE_VSA10	0.04192	20	25.12	19.81	17.42	10.54	58	87
SlogP_VSA8	0.17538	1	30.11	19.34	20.32	10.54	48	83
MollogP	0.03121	41	21.68	21.22	11.31	10.62	74	76
SMR_VSA6	0.02185	70	26.21	19.97	16.81	10.62	60	79
NumValenceElectrons	0.01197	120	22.62	17.78	14.06	10.66	76	77
PEOE_VSA4	0.02366	62	30.73	20.44	24.41	10.66	23	81
fr_nhpyrrole	0.00749	141	34.63	18.25	27.39	10.73	10	76

fr_oxime	0.01892	83	35.73	19.81	29.41	10.77	4	76
fr_nh1	0.00302	160	31.05	21.37	23.61	10.81	32	80
PEOE_VSA11	0.05144	9	26.68	19.50	19.37	10.85	59	84
PEOE_VSA14	0.01531	104	31.05	21.53	23.19	10.89	24	69
fr_piperzine	0.02390	61	35.57	20.12	28.38	10.89	8	85
CHI1	0.01492	106	21.84	19.03	13.56	10.92	80	83
SMR_VSA1	0.03495	34	21.22	17.16	12.45	11.00	80	80
fr_imidazole	0.01577	98	34.63	20.59	27.58	11.04	12	83

**LNCaP Features**	**Averaged absolute SHAP contribution**	**Ranking of features based on SHAP values**	**% of misclassified compounds with opposite-class ranges of values**		**% of correctly classified compounds with opposite-class ranges of values**		**Number of clusters with non-overlapping class values**	
			**"RAW"**	**SHAP**	**"RAW"**	**SHAP**	**"RAW"**	**SHAP**
Estate_VSA1	0.06146	33	54.29	48.00	20.94	19.37	29	33
SMR_VSA6	0.07892	19	71.43	56.57	34.15	20.16	18	29
MinPartialCharge	0.07853	20	59.43	60.57	28.44	20.38	21	26
Chi1	0.05976	35	56.57	49.14	20.27	20.60	29	29
PEOE_VSA3	0.11555	6	76.57	50.86	45.24	20.60	11	34
3D_Inertialshapefactor	0.13500	3	58.86	55.43	22.40	20.72	30	26
SlogP_VSA1	0.07663	23	71.43	57.71	37.07	20.72	17	23
VSA_ESTATE4	0.04418	52	51.43	53.71	24.08	21.16	26	25
BERTZCT	0.08466	17	53.71	54.29	22.28	21.39	33	26
SlogP_VSA5	0.14504	2	56.00	58.29	22.06	21.50	33	27
VSA_ESTATE6	0.11411	7	55.43	50.86	23.85	21.50	31	35
KAPPA2	0.03178	62	57.71	51.43	22.96	21.95	27	35
KAPPA1	0.04749	47	57.14	54.86	23.07	22.17	27	23
Chi1n	0.09545	12	56.00	55.43	22.06	22.40	29	29
fr_al_coo	0.02820	69	94.86	58.29	64.28	22.40	3	35
Chi0n	0.08313	18	56.00	58.29	22.06	22.62	29	32
SMR_VSA7	0.05798	37	58.86	53.14	29.68	22.73	22	27
AvgIpc	0.13356	4	58.29	54.86	23.52	22.84	26	25
HeavyAtomMolWT	0.02754	73	55.43	55.43	24.41	23.07	26	26
Hallkieralpha	0.02957	66	59.43	52.00	29.45	23.07	23	25

Table S10. Overall prediction performances evaluated for the "RAW" and SHAP flagging rules using different quantile global (***Panel A***) and per-cluster (***Panel B***) thresholds for misclassification reduction. Best quantiles identified for both rules setting are highlighted in bold.

Panel A

**F1 scores**	**Quantiles evaluated**							
	**80**		**85**		**90**		**95**	
	**RAW features**	**SHAP values**	**RAW features**	**SHAP values**	**RAW features**	**SHAP values**	**RAW features**	**SHAP values**
PC3	**0.308**	0.326	0.287	**0.327**	0.259	0.253	0.191	0.183
DU-145	**0.285**	0.278	0.282	**0.272**	0.247	0.252	0.209	0.214
LNCaP	0.409	**0.475**	**0.414**	0.456	0.358	0.427	0.287	0.383

Panel B

**F1 scores**	**Quantiles evaluated**							
	**80**		**85**		**90**		**95**	
	**RAW features**	**SHAP values**	**RAW features**	**SHAP values**	**RAW features**	**SHAP values**	**RAW features**	**SHAP values**
PC3	**0.285**	0.316	0.277	**0.317**	0.270	0.267	0.267	0.263
DU-145	**0.318**	0.328	0.316	**0.329**	0.314	0.327	0.314	0.325
LNCaP	0.255	**0.260**	**0.256**	0.258	0.246	0.256	0.252	0.255

Table S11. Numbers and percentages of misclassified and correctly classified compounds removed by models' predictions, at different levels of confidence.

Predict_proba	TEST SET	Number of misclassified compounds	Number of correctly classified compounds	TOTAL compounds	% of misclassified compounds	% of correctly classified compounds
≥ 50 %	PC3	758	3119	3877	19,6	80,4
≥ 60 %	PC3	494	2779	3273	15,1	84,9
≥ 70 %	PC3	285	2346	2631	10,8	89,2
≥ 80 %	PC3	136	1775	1911	7,1	92,9
≥ 90 %	PC3	34	899	933	3,6	96,4
≥ 50 %	DU-145	586	2618	3204	18,3	81,7
≥ 60 %	DU-145	419	2341	2760	15,2	84,8
≥ 70 %	DU-145	245	1960	2205	11,1	88,9
≥ 80 %	DU-145	122	1434	1556	7,8	92,2
≥ 90 %	DU-145	31	737	768	4,0	96,0
≥ 50 %	LNCaP	173	893	1066	16,2	83,8
≥ 60 %	LNCaP	133	843	976	13,6	86,4
≥ 70 %	LNCaP	101	782	883	11,4	88,6
≥ 80 %	LNCaP	63	707	770	8,2	91,8
≥ 90 %	LNCaP	27	572	599	4,5	95,5

Table S12. Percentages of misclassified and correctly classified compounds removed from models' predictions according to the proposed flagging rules, at different levels of models' confidence, for each test set.

Predict_proba	Number of misclassified compounds removed by flagging rule	Number of correctly classified compounds removed by flagging rule	TEST SET	Flagging rule	% of misclassified compounds (corrected by flagging rule)	% of correctly classified compounds (corrected by flagging rule)	Relative % of misclassified compounds removed by the flagging rule	Relative % of correctly predicted compounds removed by the flagging rule
≥ 50 %	144	237	PC3	RAW	17,6	82,4	19,0	7,6
≥ 60 %	101	197	PC3	RAW	13,2	86,8	20,4	7,1
≥ 70 %	54	150	PC3	RAW	9,5	90,5	18,9	6,4
≥ 80 %	27	82	PC3	RAW	6,0	94,0	19,9	4,6
≥ 90 %	9	31	PC3	RAW	2,8	97,2	26,5	3,4
≥ 50 %	57	124	PC3	SHAP	19,0	81,0	7,5	4,0
≥ 60 %	42	94	PC3	SHAP	14,4	85,6	8,5	3,4
≥ 70 %	28	64	PC3	SHAP	10,1	89,9	9,8	2,7
≥ 80 %	15	32	PC3	SHAP	6,5	93,5	11,0	1,8
≥ 90 %	6	8	PC3	SHAP	3,0	97,0	17,6	0,9
≥ 50 %	157	260	PC3	RAW OR SHAP	17,4	82,6	20,7	8,3
≥ 60 %	110	213	PC3	RAW OR SHAP	13,0	87,0	22,3	7,7
≥ 70 %	59	161	PC3	RAW OR SHAP	9,4	90,6	20,7	6,9
≥ 80 %	31	88	PC3	RAW OR SHAP	5,9	94,1	22,8	5,0
≥ 90 %	10	32	PC3	RAW OR SHAP	2,7	97,3	29,4	3,6
≥ 50 %	44	101	PC3	RAW AND SHAP	19,1	81,0	7,5	4,0
≥ 60 %	33	78	PC3	RAW AND SHAP	14,6	85,6	8,5	3,4
≥ 70 %	23	53	PC3	RAW AND SHAP	10,3	89,9	9,8	2,7
≥ 80 %	11	26	PC3	RAW AND SHAP	6,7	93,5	11,0	1,8
≥ 90 %	5	7	PC3	RAW AND SHAP	3,1	97,0	17,6	0,9
≥ 50 %	126	208	DU-145	RAW	16,0	84,0	21,5	7,9
≥ 60 %	86	175	DU-145	RAW	13,3	86,7	20,5	7,5
≥ 70 %	61	135	DU-145	RAW	9,2	90,8	24,9	6,9
≥ 80 %	31	87	DU-145	RAW	6,3	93,7	25,4	6,1

≥ 90 %	11	46	DU-145	RAW	2,8	97,2	35,5	6,2
≥ 50 %	127	186	DU-145	SHAP	15,9	83,9	18,3	4,9
≥ 60 %	94	150	DU-145	SHAP	12,9	86,8	18,6	4,3
≥ 70 %	65	120	DU-145	SHAP	8,9	90,9	23,3	3,9
≥ 80 %	36	78	DU-145	SHAP	6,0	93,7	23,8	3,3
≥ 90 %	13	34	DU-145	SHAP	2,5	97,3	35,5	3,1
≥ 50 %	107	127	DU-145	RAW AND SHAP	16,1	84,2	24,9	10,2
≥ 60 %	78	101	DU-145	RAW AND SHAP	13,2	87,0	24,3	9,6
≥ 70 %	57	76	DU-145	RAW AND SHAP	9,1	91,0	28,2	9,1
≥ 80 %	29	47	DU-145	RAW AND SHAP	6,3	94,0	31,1	8,2
≥ 90 %	11	23	DU-145	RAW AND SHAP	2,7	97,4	41,9	7,7
≥ 50 %	146	267	DU-145	RAW OR SHAP	15,8	84,1	21,7	7,1
≥ 60 %	102	224	DU-145	RAW OR SHAP	13,0	87,1	22,4	6,4
≥ 70 %	69	179	DU-145	RAW OR SHAP	9,0	91,1	26,5	6,1
≥ 80 %	38	118	DU-145	RAW OR SHAP	6,0	94,0	29,5	5,4
≥ 90 %	13	57	DU-145	RAW OR SHAP	2,6	97,5	41,9	4,6
≥ 50 %	84	147	LNCaP	RAW	10,7	89,3	48,6	16,5
≥ 60 %	66	135	LNCaP	RAW	8,6	91,4	49,6	16,0
≥ 70 %	53	117	LNCaP	RAW	6,7	93,3	52,5	15,0
≥ 80 %	34	103	LNCaP	RAW	4,6	95,4	54,0	14,6
≥ 90 %	16	71	LNCaP	RAW	2,1	97,9	59,3	12,4
≥ 50 %	80	123	LNCaP	SHAP	10,8	89,2	46,2	13,8
≥ 60 %	67	107	LNCaP	SHAP	8,2	91,8	50,4	12,7
≥ 70 %	49	88	LNCaP	SHAP	7,0	93,0	48,5	11,3
≥ 80 %	30	71	LNCaP	SHAP	4,9	95,1	47,6	10,0
≥ 90 %	14	42	LNCaP	SHAP	2,4	97,6	51,9	7,3
≥ 50 %	110	207	LNCaP	RAW OR SHAP	8,4	91,6	63,6	23,2
≥ 60 %	85	186	LNCaP	RAW OR SHAP	6,8	93,2	63,9	22,1
≥ 70 %	64	159	LNCaP	RAW OR SHAP	5,6	94,4	63,4	20,3

≥ 80 %	40	135	LNCaP	RAW OR SHAP	3,9	96,1	63,5	19,1
≥ 90 %	19	91	LNCaP	RAW OR SHAP	1,6	98,4	70,4	15,9
≥ 50 %	54	63	LNCaP	RAW AND SHAP	12,5	87,5	31,2	7,1
≥ 60 %	48	56	LNCaP	RAW AND SHAP	9,7	90,3	36,1	6,6
≥ 70 %	38	46	LNCaP	RAW AND SHAP	7,9	92,1	37,6	5,9
≥ 80 %	24	39	LNCaP	RAW AND SHAP	5,5	94,5	38,1	5,5
≥ 90 %	11	22	LNCaP	RAW AND SHAP	2,8	97,2	40,7	3,8

Figure S1. Distribution of the compounds in the PC3, DU-145 and LNCaP datasets, according to MACCS and ECFP4 fingerprints-based similarity estimations. For each dataset, the distribution of the compounds was evaluated by tacking a randomly selected compound as a query. In particular, CHEMBL316006, CHEMBL4067713 and CHEMBL92 were selected as queries for the similarity analyses on the PC3, DU-145 and LNCaP datasets, respectively. Red dashed lines define commonly accepted thresholds of similarity (*i.e.*, Tanimoto index MACCS = 0.8; Tanimoto index ECFP4 = 0.3).

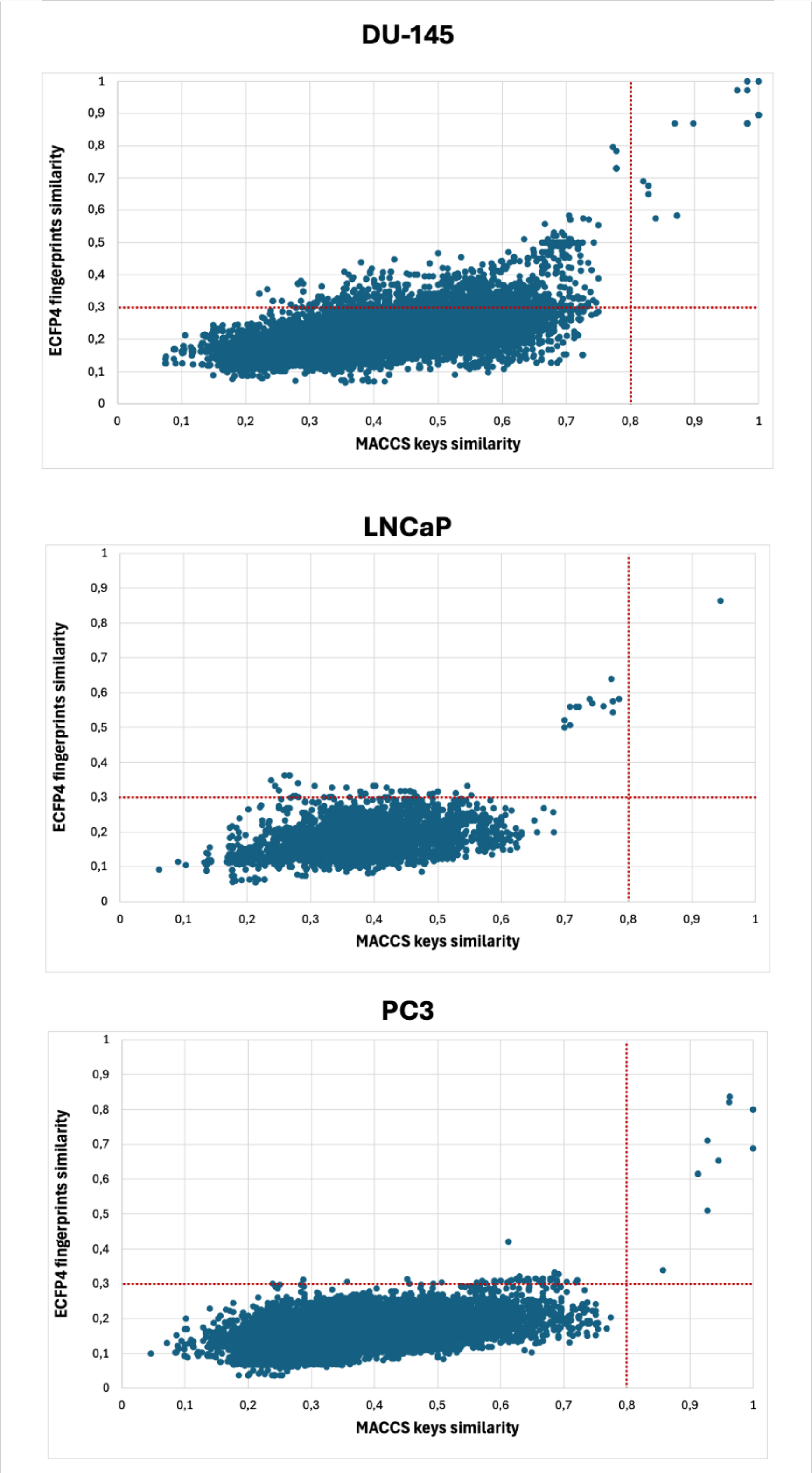

Figure S2. Pie charts displaying active and inactive compounds in the PC3, DU-145, and LNCaP datasets. Active compounds present GI_{50}, IC_{50}, EC_{50}, or ED_{50} values below 10 μM, while the inactive ones present activity values above 10 μM, or % of inhibition lower than 50% at 10μM concentration.

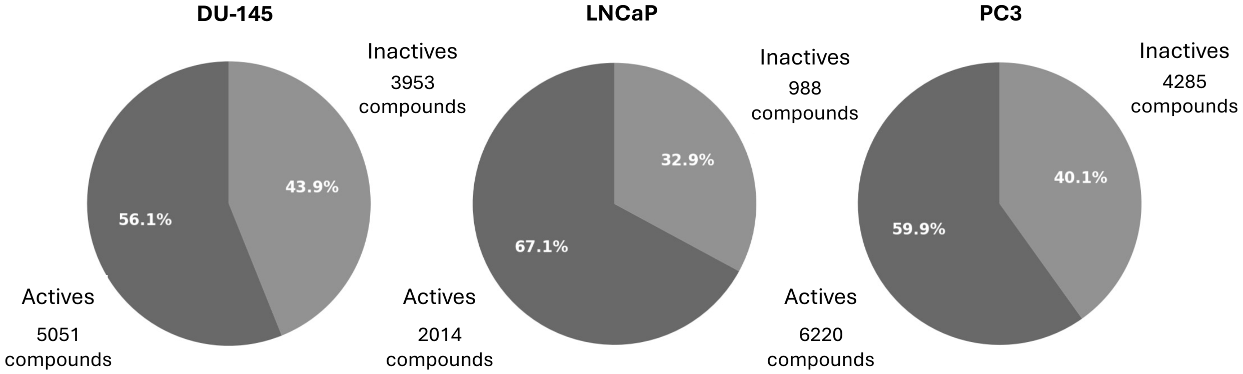

Figure S3. Comparison of training set cross-validation fold values of performance metrics; columns represent cell lines while rows represent specific performance metric evaluated.

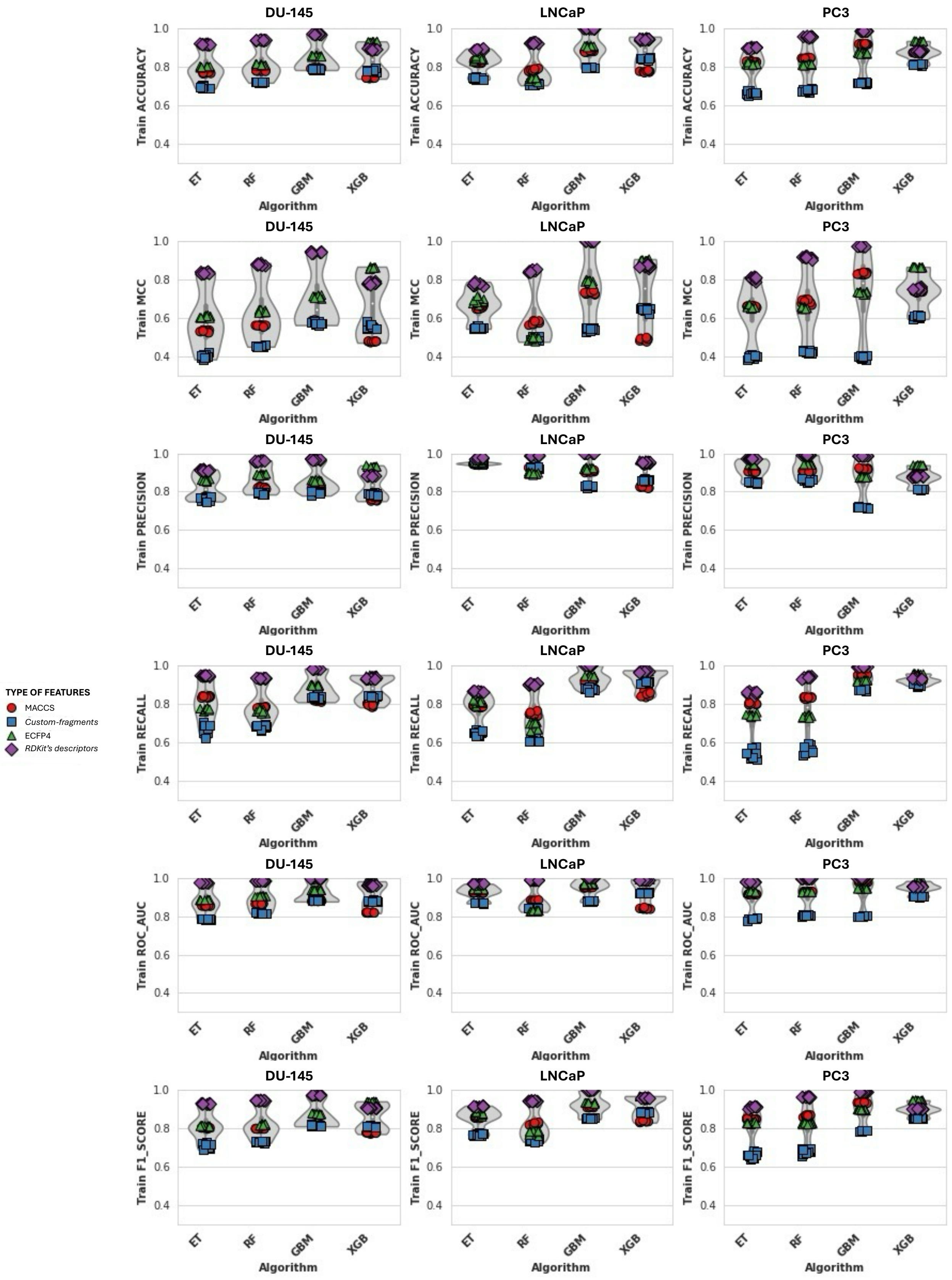

Figure S4. Averaged values of all performance metrics, aggregated *per* type of features (blue columns) or algorithm (colored columns).

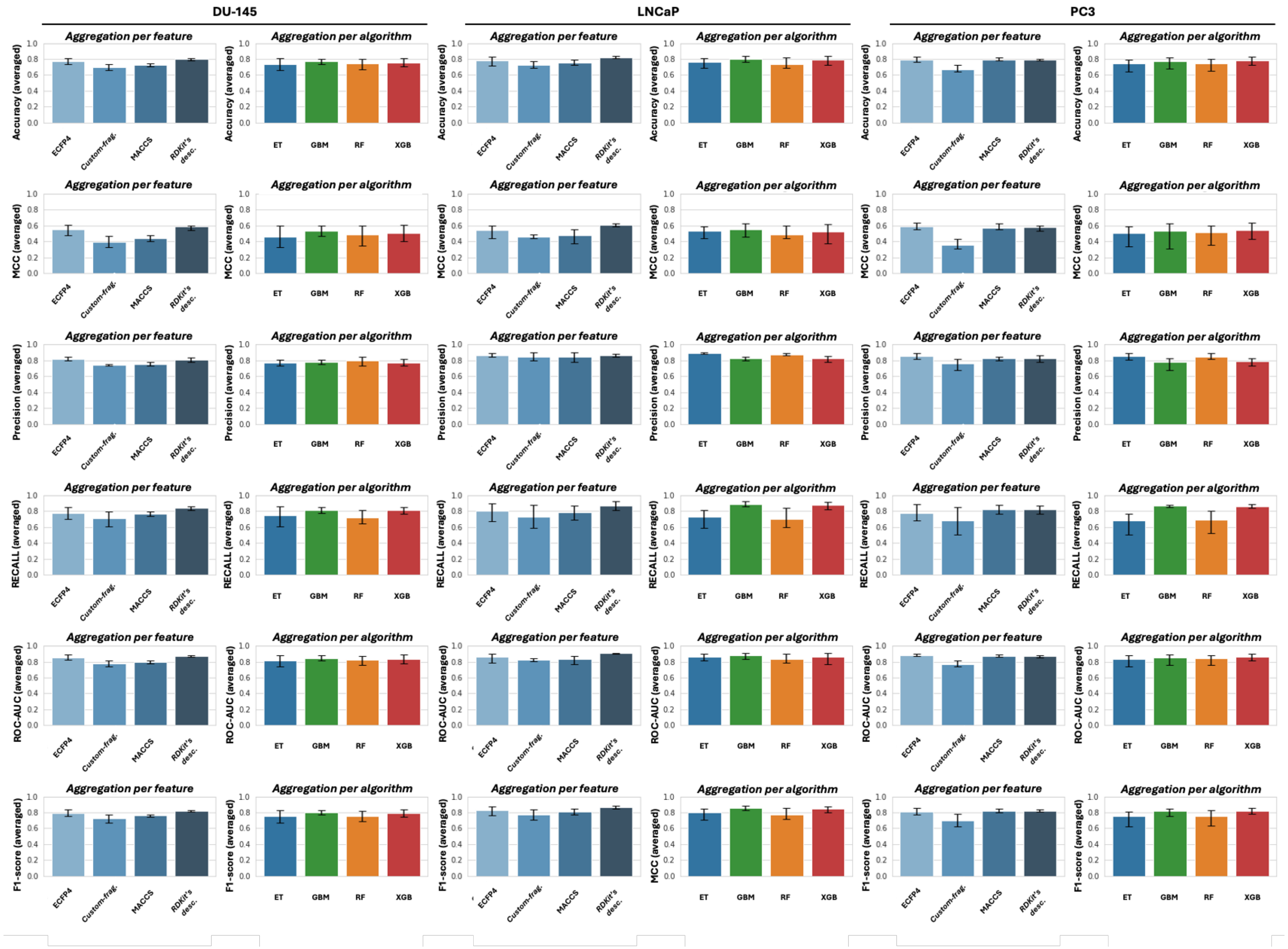

Figure S5. Percentage of features with a positive (blue columns) or null (orange columns) cumulative SHAP value contribution as evaluated for developed ML classifiers.

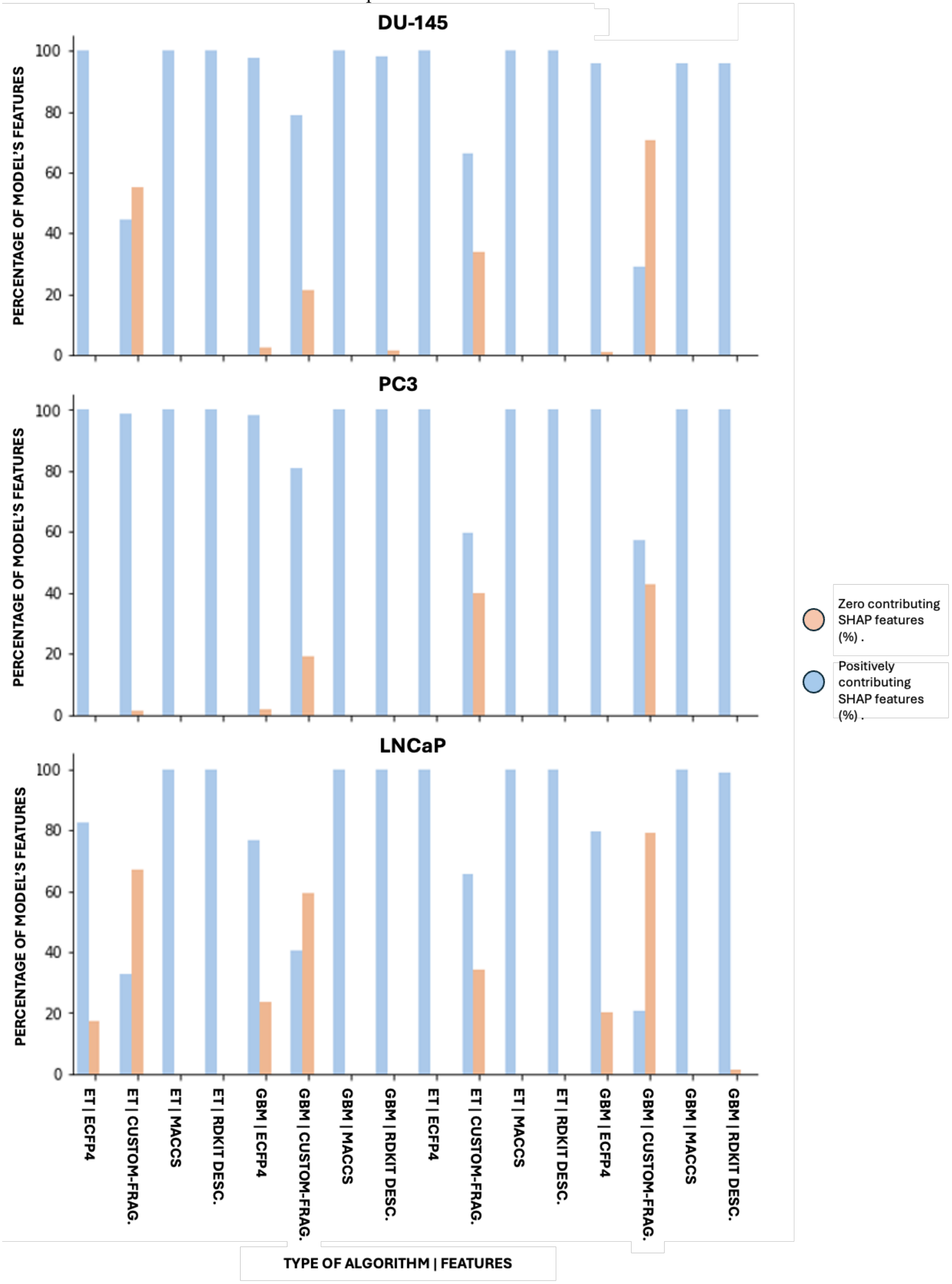

Figure S6. Distribution of the compounds in the PC3, DU-145 and LNCaP test sets, according to MACCS and ECFP4 fingerprints-based similarity estimations. For each dataset, the distribution of the compounds was evaluated by tacking a randomly selected compound as a query. In particular, CHEMBL269081, CHEMBL39541 and CHEMBL428647 were selected as queries for the similarity analyses on the PC3, DU-145 and LNCaP test sets, respectively. Red dashed lines define commonly accepted thresholds of similarity (i.e., Tanimoto index MACCS = 0.8; Tanimoto index ECFP4 = 0.3).

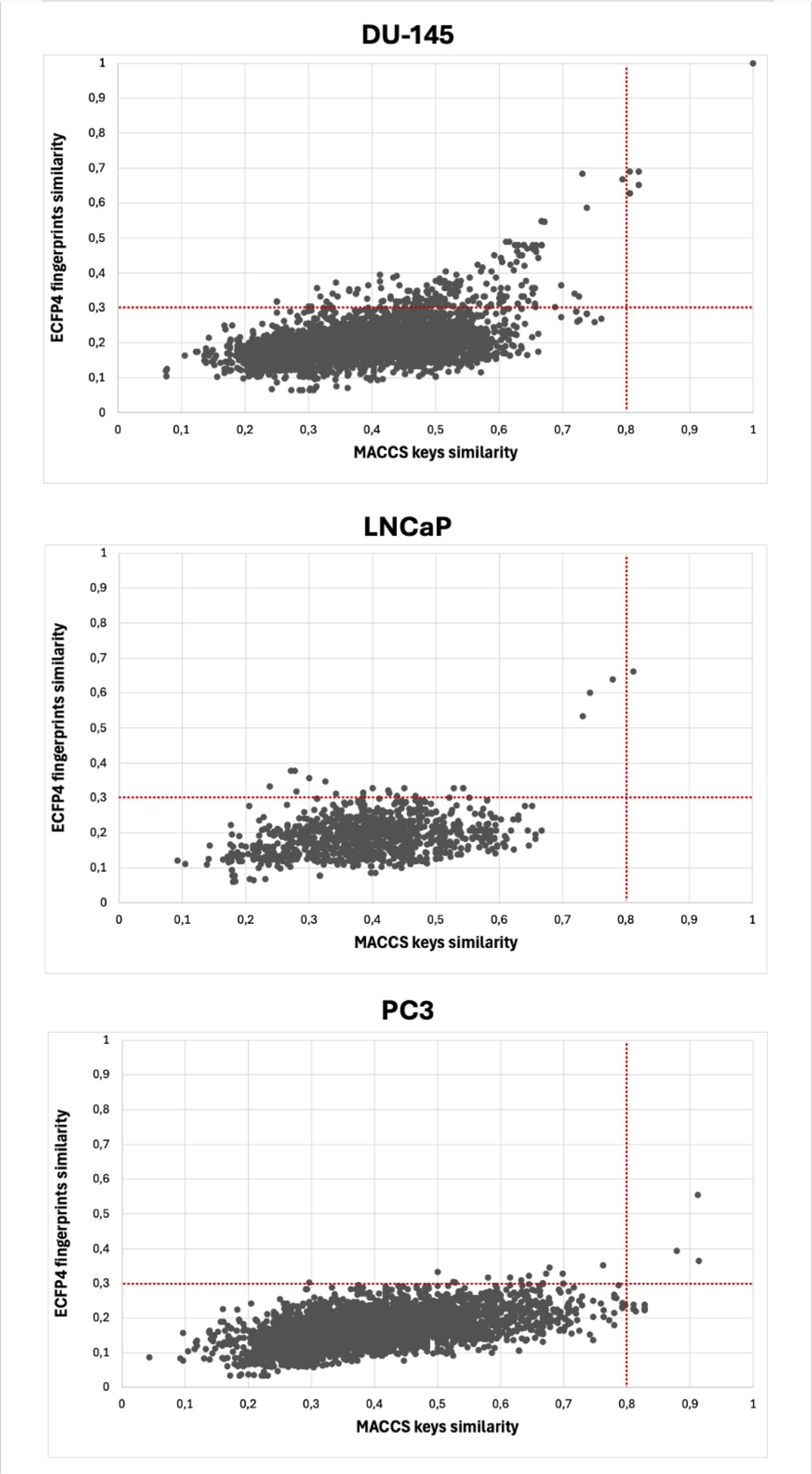

Figure S7. Percentage of molecules in the PC3 dataset with "RAW" or SHAP values in the ranges of the opposite class of prediction, for the misclassified and the correctly classified molecules populations, considering the top20 contributing features by mean SHAP values.

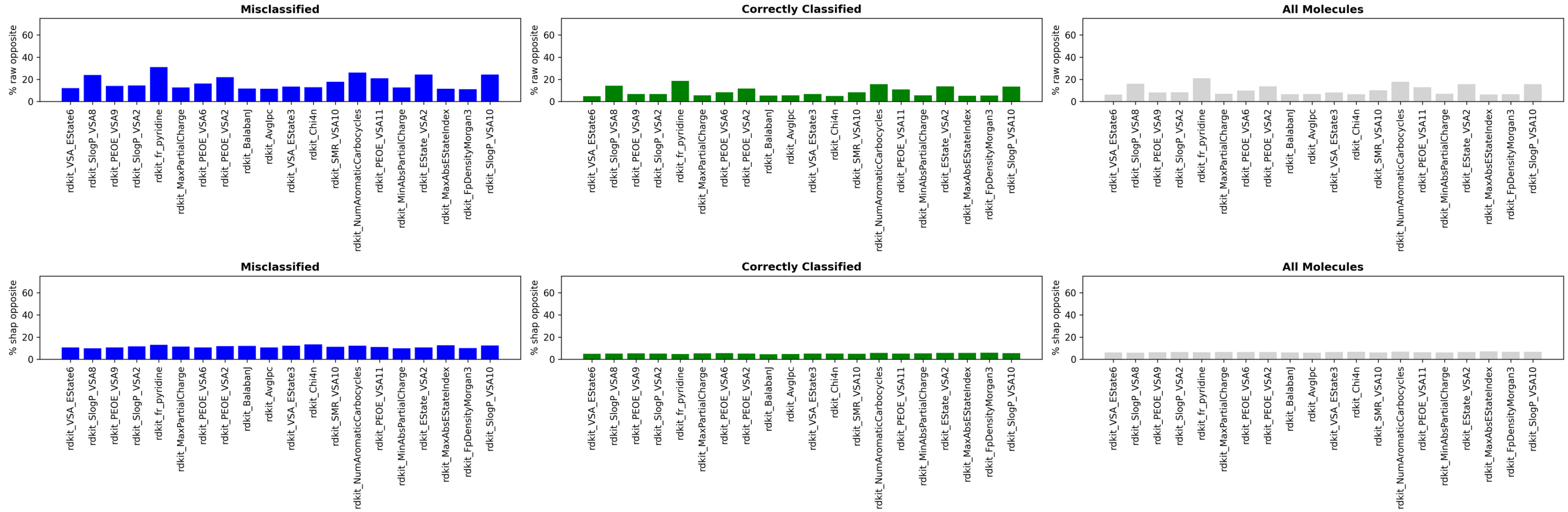

Figure S8. Percentage of molecules in the DU-145 dataset with "RAW" or SHAP values in the ranges of the opposite class of prediction, for the misclassified and the correctly classified molecules populations, considering the top20 contributing features by mean SHAP values.

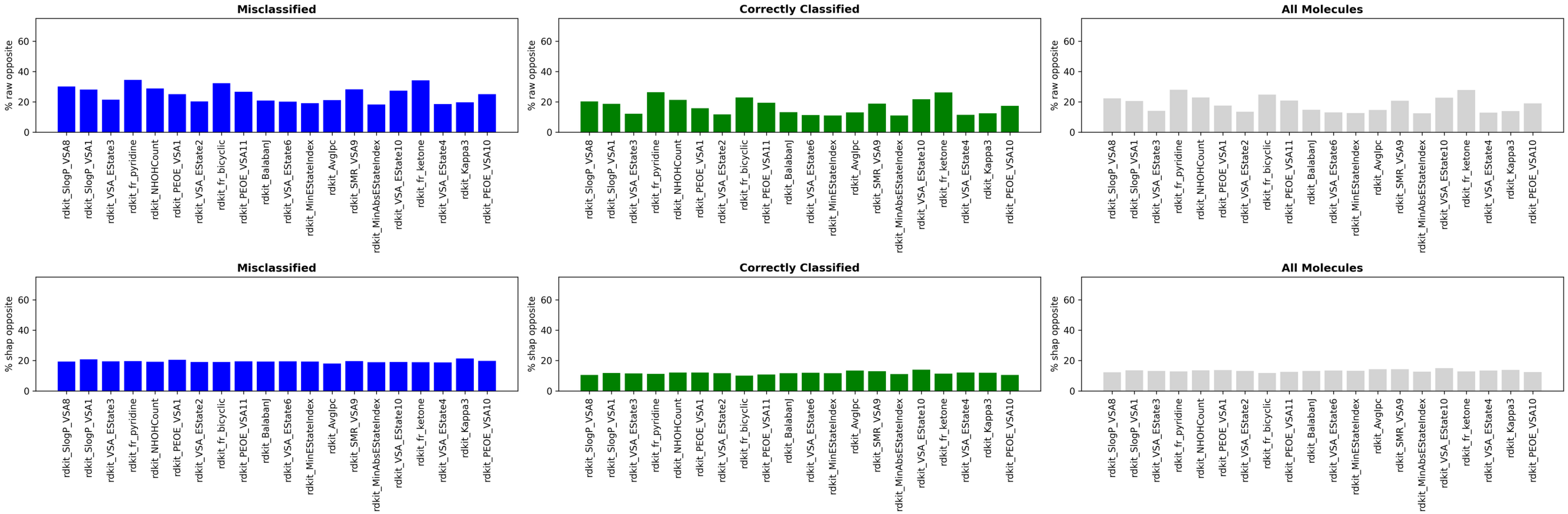

Figure S9. Percentage of molecules in the LNCaP dataset with "RAW" or SHAP values in the ranges of the opposite class of prediction, for the misclassified and the correctly classified molecules populations, considering the top20 contributing features by mean SHAP values.

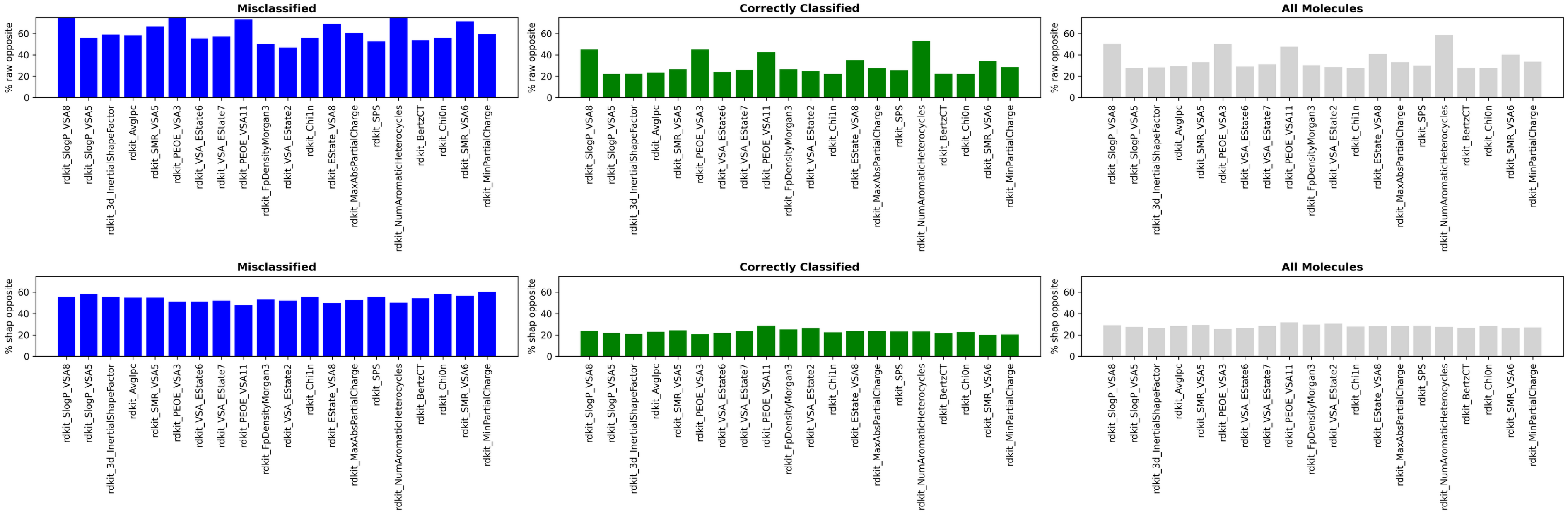

Supplement: Supplementary file 1 [file ci5c02015_si_001.pdf]
